# Peptide-functionalized nanoparticles for brain-targeted therapeutics

**DOI:** 10.1007/s13346-025-01840-w

**Published:** 2025-03-31

**Authors:** Sophia Tang, Emily L. Han, Michael J. Mitchell

**Affiliations:** 1 Department of Bioengineering, School of Engineering and Applied Science, University of Pennsylvania, Philadelphia, PA 19104 USA; 2 Abramson Cancer Center, Perelman School of Medicine, University of Pennsylvania, Philadelphia, PA 19104 USA; 3 Center for Cellular Immunotherapies, Perelman School of Medicine, University of Pennsylvania, Philadelphia, PA 19104 USA; 4 Penn Institute for RNA Innovation, Perelman School of Medicine, University of Pennsylvania, Philadelphia, PA 19104 USA; 5 Institute for Immunology, Perelman School of Medicine, University of Pennsylvania, Philadelphia, PA 19104 USA; 6 Cardiovascular Institute, Perelman School of Medicine, University of Pennsylvania, Philadelphia, PA 19104 USA; 7 Institute for Regenerative Medicine, Perelman School of Medicine, University of Pennsylvania, Philadelphia, PA 19104 USA

**Keywords:** Peptides, Blood–Brain Barrier, Nanoparticles, Drug Delivery

## Abstract

Despite the rapid development of nanoparticle (NP)-based drug delivery systems, intravenous delivery of drugs to the brain remains a major challenge due to various biological barriers. To achieve therapeutic effects, NP-encapsulated drugs must avoid accumulation in off-target organs and selectively deliver to the brain, successfully cross the blood–brain barrier (BBB), and reach the target cells in the brain. Conjugating receptor-specific ligands to the surface of NPs is a promising technique for engineering NPs to overcome these barriers. Specifically, peptides as brain-targeting ligands have been of increasing interest given their ease of synthesis, low cytotoxicity, and strong affinity to target proteins. The success of peptides as targeting ligands is largely due to the diverse strategies of designing and modifying peptides with favorable properties, including membrane permeability and multi-receptor targeting. Here, we review the design and implementation of peptide-functionalized NP systems for neurological disease applications. We also explore advances in rational peptide design strategies for brain targeting, including using generative deep-learning models to computationally design new peptides.

**Graphical Abstract:**

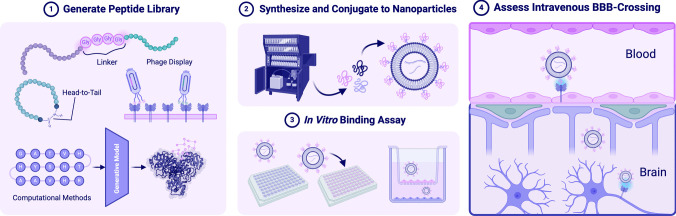

## Introduction

The development of non-invasive therapeutics that can be administered intravenously remains a challenge for the treatment of neurological disorders, including glioma, Alzheimer’s disease (AD), and Parkinson’s disease (PD), among others [1]. Nanoparticles (NPs) are a powerful vehicle for drug delivery, specifically for large macromolecules including RNA therapeutics and CRISPR/Cas9 gene-editing proteins [2]. The advantages of NP encapsulation of therapeutics include prolonged circulation times, efficient transport through cell membranes via endocytosis, controlled release in target tissue, efficient drug loading, and reduced side effects [3–5]. Despite the significant advantages of NPs, there remain limitations in delivery and biodistribution to target tissues due to natural clearance to the liver and spleen and the presence of biological barriers [2]. One of the most restrictive biological barriers in the human body is the blood–brain barrier (BBB), a semi-permeable barrier that prevents ~ 98% of small molecule drugs and ~ 100% of macromolecule drugs from entering the brain parenchyma from the bloodstream [6]. The BBB consists of a layer of brain capillary endothelial cells (BCECs) connected by tight junctions, astrocytes, pericytes, and neurons, allowing only selected molecules to cross [1, 7].

Due to their relatively large size, NPs primarily enter the brain via transcytosis across the brain endothelium. One method of increasing transcytosis is with active targeting, in which ligands with high affinity for specific receptors are conjugated to NPs to facilitate receptor-mediated transcytosis (RMT). In RMT, the targeting ligands mediate the binding of NPs to receptors on the surface of BCECs, inducing the formation of endosomes that engulf the NPs and traffic them through the cell for subsequent release into the brain parenchyma via exocytosis [1]. However, several challenges, including unpredictable uptake routes, removal by efflux transporters, and failure to exocytose at the abluminal side of the brain endothelium, can inhibit the efficacy of RMT via active targeting [8, 9]. Furthermore, NPs may encounter acidic degradation and lysosomal recycling during transcytosis, which poses additional challenges for NPs containing therapeutic cargoes with intracellular mechanisms such as gene editing and nucleic acid therapies [9–12].

**Fig. 1:**
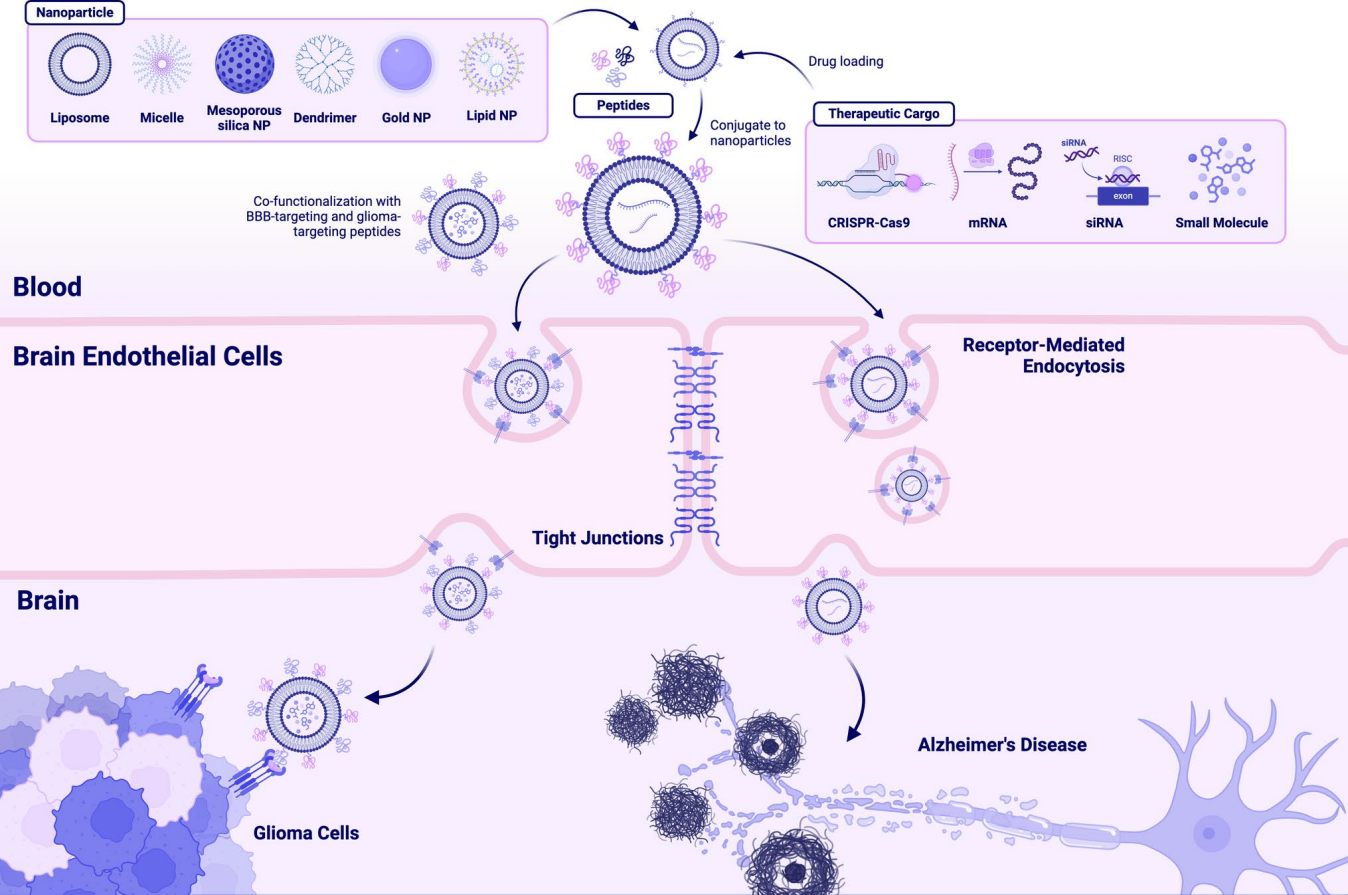
Peptide-functionalized nanoparticles (NPs) for targeted therapeutic delivery across the blood–brain barrier (BBB). Overview schematic depicting different types of NPs carrying diverse therapeutic cargos that are functionalized with peptides. These peptides target receptors overexpressed on brain capillary endothelial cells that comprise the BBB, facilitating receptor-mediated endocytosis and subsequent trafficking into the brain parenchyma. These targeted NPs can then bind to receptors overexpressed on disease cell types of interest such as glioma cells

Several classes of ligands have been explored for BBB-targeting and crossing, including antibodies, proteins, peptides, and aptamers that specifically bind to receptors abundant on the BBB. Peptides have several properties that make them optimal ligands for surface-functionalization. These include small molecular weights, easy synthesis, and low cytotoxicity and immunogenicity [13]. In addition, peptides are a robust platform for rational engineering, enabling diverse backbone and sidechain modifications, cyclizations, and conjugation with other chemical groups (Fig. 1).

This review covers the space of well-established brain-targeting peptides that have been conjugated to a diverse array of NPs for intravenous delivery of drugs for neurological diseases, highlighting results that demonstrate the promise of peptides for BBB-specific targeting and crossing. Further, we discuss the class of cell-penetrating peptides used to enhance drug delivery into cells. We review rational design strategies for integrating peptides into NP-delivery systems and enhancing the peptides' properties — from introducing chemical modifications to fusing multiple peptides. Finally, given that the majority of receptors expressed on diverse cell types and disease phenotypes in the brain have no existing peptide binders, we highlight the emerging field of de novo peptide design through generative deep-learning models. This review aims to advance the development of NP-based precision therapeutics for neurological disorders through the rational design of delivery vehicles that can be repurposed for efficient delivery of any therapeutic cargo across the BBB.

## Brain-targeting peptides

Peptides such as RVG29, T7, Angiopep-2 (Ang), and mApoE have emerged as critical tools for facilitating the delivery of NP-encapsulated therapeutics across the BBB. These peptides, which bind to recpetors highly expressed on BCECs or neuronal cell surfaces, have been extensively studied for their ability to enhance BBB crossing (Table 1). Functionalization of NPs with these peptides has demonstrated substantial improvements in drug delivery efficacy, enabling transport across the BBB and enhanced cellular uptake. In particular, peptide-conjugated NPs have shown promise in achieving effective cytoplasmic delivery for applications such as gene knockdown and nucleic acid-based gene editing. These peptides not only serve as a foundation for optimizing surface-conjugation strategies and developing dual-targeting formulations but also drive the rational design of next-generation delivery peptides.

**Table 1:** Brain-targeting peptides and their applications in functionalizing nanoparticles (NPs) for delivery of various cargo. Leftmost column also indicates with a “ + ” notation any additional targeting ligands used on the nanocarrier

Peptide	Sequence	Target Receptor(s)	Disease and Cargo	Nanocarrier	Citation
RVG29	YTIWMPENPRPGTPCDIFTNSRGKRASNG	nAchR and GABA receptors	ApoE2 (apolipoprotein E isoform) encoding plasmid DNA (pApoE2) for Alzheimer’s disease	Liposomes	[14]
			Dopamine derivative N-3,4-bis(pivaloyloxy)-dopamine (BPD) for Parkinson’s Disease	PEGylated liposomes	[15]
			miR-124 for Parkinson’s Disease	Polymeric nanoparticles	[16]
			Docetaxel (DTX) for glioma	PEG-PLGA NPs	[17]
			Genistein (GS) for Alzheimer’s disease	Macrophage membrane-coated solid lipid nanoparticles (SLNs)	[18]
			Beta-site amyloid precursor protein cleaving enzyme 1 (BACE1) siRNA for Alzheimer’s disease	Exosomes	[19]
			Luciferase and mCherry mRNA	Ionizable Lipid Nanoparticles (LNPs)	[20]
T7	HAIYPRH	Tf receptor (TfR)	siRNA for glioma	Dendrigraft poly-l-lysines (DGLs)	[21]
			cediranib (CD) and paclitaxel (PTX) for glioma	PEGylated bilirubin nanoparticles (BRNPs)	[22]
			Antisense miRNA oligonucleotides against miR-21 (AMO-21) for glioblastoma	Exosomes	[23]
			Carmustine for glioma	PEG-PLGA Micelles	[24]
+ Tet1 peptide			BACE1 siRNA for Alzheimer’s disease	DGL-based nanoparticle	[25]
+ stroke homing peptide (SHp)			Neuroprotectant (ZL006) for stroke therapy	Liposome	[26]
			Vincristine sulfate (VCR) anti-tumour drug for glioma	Low-density lipoprotein (LDL) particles	[27]
Angiopep-2 (Ang)	TFFYGGSRGKRNNFKTEEY	LDLR-related protein 1 (LRP1)	Cisplatin (CDDP) for human glioblastoma multiforme (GBM)	Layer-by-layer (LbL)-NPs consist of a charged NP core and polyelectrolyte multilayer shell	[28]
+ tumor-homing neurophilin-1 receptor peptide			Vascular endothelial growth factor (VEGF) siRNA and docetaxel (DTX) for glioma	Liposomes	[29]
			Tumor necrosis factor-related apoptosis-inducing ligand (TRAIL) gene therapy for glioma	Polyamidoamine dendrimer (PAMAM) NPs	[30]
+ TAT cell-penetrating peptide			Docetaxel (DTX) for glioma	Tandem nanomicelles and small extracellular vesicles (sEVs)	[31, 32]
+ activatable cell-penetrating peptide (ACP)			DTX for glioma	PEG-PCL NPs	[33]
	Retro-inverso sequence		Glioma therapy	PEG-DSPE micelles	[34]
+ EGFP-EGF1 protein targeting neuroglial cells			Neuroglial-related disease	PEG-PCL NPs	[35]
mApoE	RLLRKRLKRLGW	LDLR and LDLR-related proteins 1 and 2 (LRP1 and LRP2)	^—^	Solid lipid nanoparticles (SLNs)	[36]
+ CITx			Doxorubicin (DOX) for glioblastoma (CITx highly expressed on tumour cells)	Liposomes	[37, 38]
+ phosphidic acid			Aβ plaque reduction for Alzchmimer’s disease	Liposomes	[39–41]

### RVG29 peptides

RVG29 is a 29-amino acid peptide derived from rabies virus glycoprotein that binds specifically to the nicotinic acetylcholine receptor (nAchR), expressed on BCECs and neurons, and gamma-aminobutyric acid (GABA) receptors also expressed on neurons [13]. Inspired by the ability of neurotropic viruses like the rabies virus to overcome the BBB in vivo, Kumar et al. first showed that RVG29 could specifically bind to Neuro2a neuronal cells expressing nAchR in vitro and enter the mouse brain in vivo [42]. After conjugation with free small interfering RNA (siRNA), RVG29 also facilitated specific gene silencing in the brain after intravenous injection into GFP transgenic mice, indicating that RVG29 not only facilitates BBB crossing but also brain cell transfection in vivo [42]. Given these promising results, RVG29 has since been conjugated onto various NP-based systems to enhance bioavailability and serum half-life, including poly amidoamine dendrimers [13], liposomes [14, 15], solid lipid nanoparticles [18], and ionizable lipid nanoparticles [20] for delivery of various cargoes. Notably, Han et al. [20] showed that RVG29-conjugated ionizable lipid nanoparticles (LNPs) increased BBB-crossing and transfection of luciferase mRNA in the brain by ~ 70-fold after intravenous injection to healthy adult mice [20]. Furthermore, flow cytometry analysis showed that RVG29 LNPs encapsulating mCherry mRNA resulted in an increase in neuron transfection from ~ 1.2% to ~ 2.4% and no increase in endothelial cell transfection, indicating that RVG29 can facilitate the crossing of mRNA across the BBB into the brain parenchyma [20].

Compared to healthy cells, there is a twofold increase of nAchR expression on glioma cells, allowing RVG29-conjugated NPs to cross the BBB and specifically deliver to the glioma region [17]. Hua et al. demonstrated that RVG29-conjugated PEG-PLGA NPs increased in vivo brain concentration of docetaxel by 2.1-fold and prolonged survival time by 7 days compared to unmodified NPs in a rat model [17].

Neurons are targets for treating AD due to the expression of beta-site amyloid precursor protein cleaving enzyme-1 (BACE1) that promotes neuronal death and cognitive decline [43]. Since RVG29 targets nAchRs and GABA receptors abundant on the surface of neurons, several studies have investigated RVG29-functionalized NPs for AD treatment [14, 18, 19]. Alvarez-Erviti et al. found that RVG29-conjugated exosomes enhanced BACE1 siRNA delivery to neurons, microglia, oligodendrocytes, and their precursors and resulted in 62% protein knockdown in brain tissue after intravenous injection in C57BL/6 mice [19]. Another therapeutic strategy for AD involves delivering antioxidants to the mitochondria of neurons to reduce oxidative stress, which is related to the production of Aβ. Han et al. developed dual-functionalized solid-lipid nanoparticles (SLNs) with RVG29 for BBB- and neuron-targeting and positively-charged triphenylphosphine cation (TPP) for specific delivery to negatively-charged neuronal mitochondria after internalization. This approach facilitated a higher reduction in reactive oxygen species (ROS) and apoptosis rate in neuronal HT22 cells in vitro compared to monofunctionalized SLNs, aligning with in vivo results in a AD mouse model [18].

Since Parkinson’s disease (PD) is a result of the loss of dopaminergic neurons in the substantia nigra [44], RVG29 has also been conjugated to liposomes, polymeric nanoparticles, and exosomes to target neurons for the treatment of PD [15, 16, 45]. Notably, Qu et al. conjugated RVG29 to liposomes that effectively delivered a dopamine derivative BPD across the BBB to the striatum and substantia nigra for the treatment of PD, leading to a greater increase in dopamine levels compared to the free drug in a PD mouse model [15].

### T7 peptides

T7 is a 7-amino acid peptide that binds specifically to the transferrin (Tf) receptor (TfR) expressed on BCECs and glioma cells, binding with an affinity similar to endogenous Tf [46]. In addition, the binding site of T7 is distinct from endogenous Tf; therefore, high concentrations of endogenous Tf do not inhibit binding and can promote enhanced binding of T7 [47, 48]. T7 was first discovered by Lee et al. through phage display and was found to facilitate the internalization of green fluorescent protein (GFP) into cells expressing TfR [46].

TfR is overexpressed in glioma cells and glioma stem cells due to their role in cell metabolism and proliferation; therefore, T7 has been widely explored for the delivery of glioma and glioblastoma therapies, including siRNA, antisense microRNA (miRNA), and small molecule drugs [21–23, 27]. Notably, T7 enables enhanced brain penetration by 7.89-fold when conjugated onto PEGylated bilirubin NPs (BRNPs) and facilitated 74.1% apoptosis of C6 glioma cells after incubation in vitro, which aligned with the overexpression of TfR during C6 cell proliferation [22]. In addition, T7-conjugated BRNPs loaded with cediranib (CD) and paclitaxel (PTX) for glioma therapy prolonged survival time in a glioma mouse model by 10 days over untargeted NPs, signifying the crucial role of T7 in brain delivery of the drugs [22]. Furthermore, T7-functionalized NPs have been explored for the delivery and transfection of RNA therapies. Specifically, T7-decorated exosomes 30 to 100 nm in diameter were loaded with antisense miRNA oligonucleotides against miR-21 (AMO-21) for glioblastoma therapy, reducing tumour sizes by more than 50% in an intracranial glioblastoma rat model [23]. Since AMO-21 requires binding to the miRNA in the cell cytosol to inhibit activity, this suggests that T7 is effective in facilitating its transport across the BBB and transfection of target cells.

T7 has also been explored for the targeted delivery of the neuroprotectant drug ZL006 for stroke therapy. T7-conjugated PEGylated liposomes were able to significantly decrease ischemia-induced infarct volume and neurological deficit in a middle cerebral artery occlusion (MCAO) rat model [49]. To enhance targeting effects, Zhao et al. functionalized liposomes with T7 and stroke homing peptide (SHp) (CLEVSRKNC) that selectively accumulates in the ischemic region of mice due to excessive release of glutamate acid during the ischemic phase of the stroke [26]. The dual-targeted liposomes were able to significantly decrease ischemia-induced neural cell death, as indicated by an 11.3% reduction in TUNEL-positive cells [26].

Among the widely explored brain-targeted peptides, T7 has the shortest amino acid sequence length, preventing significant increases in NP size and enabling higher density conjugation onto the NP surface to strengthen binding affinity. In addition, TfR is overexpressed over 100-fold in cancer cells, making it an effective ligand for glioma-targeting therapies [46]. However, given the expression of TfR in almost all tissues, including the liver and lung, future work should focus on dual-functionalization of NPs systems with T7 in addition to other brain-specific targeting ligands or enhancing the brain-specificity of T7 with rational design [50].

### Angiopep-2 peptides

Angiopep-2 (Ang) is a 19-amino acid peptide that targets low-density lipoprotein receptor-related protein-1 (LRP1) highly expressed on the BBB and glioma cells [30]. Given the tenfold increase in transport of the protein aprotinin across the BBB compared to BBB-permeable proteins, including Tf, Demeule et al. derived a library of peptides from the region responsible for transport, which was identified using multiple sequence alignment to discover homologous regions in the protein which are likely affect protein function [51]. From the derived peptides, Ang showed a sevenfold increase in transcytosis compared to aprotinin in an in vitro BBB model. This prompted the conjugation of Ang to various NP-delivery systems for BBB and brain-targeted therapies.

In addition to BBB penetration, Ang can facilitate enhanced localization within neuroglial cells involved in various central nervous system (CNS) disorders including AD, PD, stroke, pain, and epilepsy. Huile et al. designed PEG-PCL NPs functionalized with Ang for BBB targeting and EGFP-EGF1 protein for the targeting of neuroglial cells [24]. This approach significantly increased NP uptake in neuroglial cells by 1.41-fold and bEnd.3 BCECs by 2.08-fold compared to unmodified NPs and displayed enhanced colocalization with neuroglial cells in an ex vivo rat model compared to Ang singly functionalized NPs.

With LRP1 highly expressed on the BBB and glioma cells, several studies have conjugated Ang to various NPs, including polyamidoamine dendrimers (PAMAM) [30], PEG-PCL NPs [33, 35], liposomes [29], tandem nano micelles [31], and small extracellular vesicles (sEVs) [32] for glioma targeting. Straehla et al. designed a novel BBB-glioblastoma (GBM) microfluidic model consisting of a tumour spheroid embedded into a self-assembled vascular endothelial layer modelling the BBB [28]. Ang-conjugated NPs showed significantly higher permeability in the BBB-GBM model compared to the non-tumor BBB model, indicating a higher abundance of LRP1 in tumor-surrounding microvessels [28]. Dual functionalization of sEVs with Ang and cell-penetrating peptide TAT has been shown to facilitate BBB transport, followed by glioma targeting and penetration in mice [29]. Due to synergistic BBB targeting and cell penetration, the dual-functionalized sEVs showed twofold and threefold stronger signals in the brain and tumour region in vivo compared to singly-modified Ang and TAT sEVs respectively, which translated into the suppression of glioma growth and increase in survival time [29].

Although Ang has BBB and glioma cell-targeting properties, deeper penetration into the tumour structure remains a barrier to therapeutic efficacy. To circumvent this, Gao et al. proposed a dual-stage glioma targeting NP functionalized with Ang and an activatable cell-penetrating peptide (ACP) to deliver docetaxel (DTX) to the glioma site [33]. The ACP used in this study is composed of an anionic E8 sequence with a cationic R8 sequence linked by sequence (PLGLAG) that is a substrate of matrix metalloproteinase-2 (MMP-2) highly expressed on C6 glioma cells. Due to electrostatic interactions between E8 and R8, the cell-penetrating properties of R8 are inhibited. First, Ang facilitates the transport of the NPs across the BBB to glioma cells via LRP1-mediated endocytosis. MMP-2 detaches the E8 sequence from the NP at the glioma site, leaving the cationic R8 peptides to facilitate penetration into glioma cells. The bifunctionalized NPs showed superior localization at the glioma region in Balb/c mice compared to monofunctionalized NPs [33]. In total, Ang serves as a versatile ligand that significantly enhances BBB-targeting in both single-ligand and dual-ligand NPs.

### mApoE peptides

mApoE is a 12-amino acid peptide and a fragment of human Apolipoprotein E (ApoE) with a high binding affinity towards all three low-density lipoprotein (LDL) receptors, including LDLR and LDLR-related proteins 1 and 2 (LRP1 and LRP2) overexpressed on BCECs compared to other tissue and glioma cells [52]. ApoE is a serum protein that facilitates the transport of lipids from the bloodstream into the CNS via LDL receptors [53, 54]. From ApoE, a tandem dimer repeat of amino acids 141–155 of ApoE, called dApoE, was shown to retain the binding affinity to LDLR [55]. Following the discovery of dApoE, Re et al. compared the uptake of dApoE and its constituent monomer sequence mApoE conjugated liposomes in BCECs and found that mApoE exhibited higher uptake at both low and high surface densities [54]. This study prompted the functionalization of various NPs with mApoE for BBB-targeting and crossing.

Several studies have co-decorated liposomes with mApoE and phosphatidic acid (PA) for binding to β-amyloid peptides (Aβ) abundant in AD [39–41]. Aimed to destabilize insoluble Aβ aggregates, Bana et al. demonstrated that liposomes co-decorated with mApoE and PA were capable of disaggregating Aβ plaques in a time- and dose-dependent manner [41]. In contrast, single-functionalized liposomes showed no effect due to the synergistic electrostatic interactions between the positively charged mApoE peptide and negatively-charged PA with differently charged residues in Aβ peptide [41]. This approach yielded a 34% reduction in the size of Aβ plaques in the hippocampus and cortex, a 33% reduction in total brain insoluble Aβ1–42, and a 70.5% reduction in brain Aβ oligomers in transgenic mice, which strongly correlates to synaptic dysfunction and disease severity [39]*.* At the same time, mono-functionalized liposomes showed no significant effect [39].

Although mApoE-functionalized liposomes showed a tenfold increase in endothelial permeability in a hCDMEC/D3 transwell model, Formicola et al. demonstrated that dual functionalization with mApoE and CITx peptide could further enhance endothelial permeability by 30-fold compared to unmodified liposomes [37]. CITx has been shown to interact with Annexin A2 involved in the exocytosis of pathogens across the BBB, suggesting that bifunctionalized liposomes undergo LDL-receptor mediated endocytosis via mApoE and enhanced exocytosis via CITx binding to Annexin A2.

Despite the success of mApoE, dApoE has also been conjugated to liposomes and chimeric polymersomes for targeting LDLR, LRP1, and LRP2 [52, 56]. Compared to mApoE, dApoE showed lower cellular uptake into endothelial cells (RBE4 cells) but higher endothelial permeability across the cell monolayer, suggesting that dApoE reduces accumulation in the endothelial layer and facilitates transport across the BBB [54]. In comparison to five previously reported BBB-penetrating peptides (Angiopep-2, CDX, HAI, SynB1, and Tat), dApoE-functionalized liposomes showed superior brain accumulation in mice, with 3.9-fold higher accumulation than unmodified liposomes [56].

Given that LDLRs are upregulated in glioblastoma development, ApoE can act as a dual-function ligand for targeting the BBB and delivery to GBM cells [52]. Functionalization of chimeric polymersomes (CPs) with ApoE peptides enhanced BBB transport in vitro by 4.8-fold compared to unmodified CPs and 2.2-fold compared to Ang-functionalized CPs, likely due to interaction with multiple receptors in the LDLR family. ApoE-CPs loaded with saporin also showed deep penetration into tumours in GBM-bearing mice and completely inhibited disease-related weight loss [52].

## Cell-penetrating peptides

**Fig. 2:**
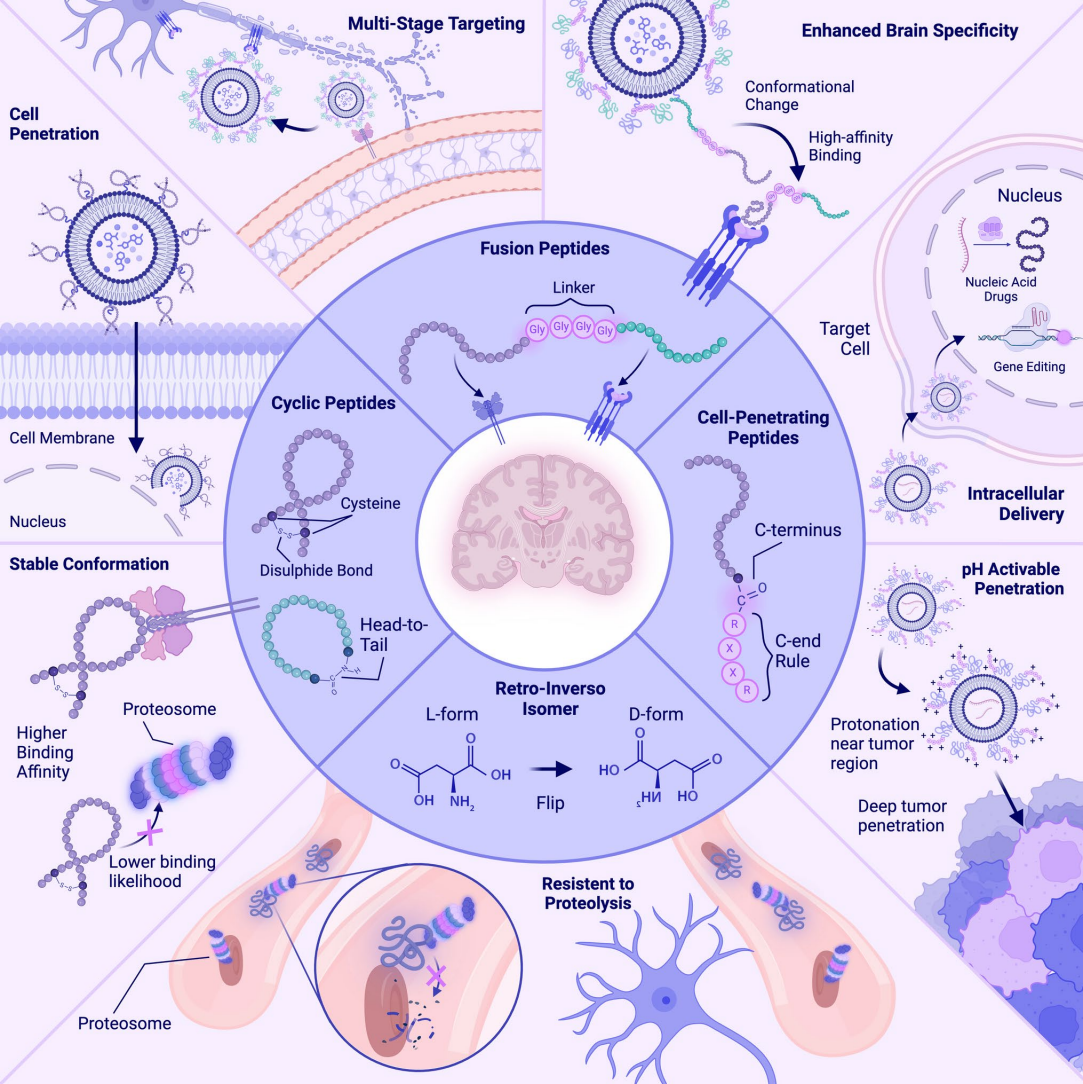
Rational design strategies to enhance peptide targeting efficacy. Schematic depicting various strategies to enhance peptide targeting for nanoparticle delivery to the brain, including designing fusion peptides with specialized linkers, utilizing cell-penetrating peptides, altering the stereochemistry of peptides, and cyclizing peptides. The inner circle shows the design strategies and the outer circle highlights the specific ways in which these strategies increase brain-targeting efficacy

Since a large portion of therapeutics — including nucleic acid and gene editing therapeutics — need to enter the cell's cytosol to achieve therapeutic effects, targeting the BBB is often insufficient. Therefore, various studies have explored the functionalization of NPs with a class of peptides that can penetrate the cell membrane called cell-penetrating peptides (CPPs) (Fig. 2). CPPs are short 5–30 amino acid peptides derived from proteins carrying protein transduction domains that can penetrate the cell membrane. Most CPPs are positively charged at physiological pH due to arginine (R) and lysine (K) residues [57, 58]. CPPs can cross lipid bilayers through receptor-mediated transcytosis, adsorptive-mediated transcytosis, or direct diffusion [59].

### Cell-penetrating peptide-functionalized nanoparticles

Given their ability to overcome receptor saturation and maintain stable cellular uptake over time, various studies have co-functionalized NPs with CPPs alongside brain-specific targeting ligands [31, 32]. TAT (GRKKRRQRRRPPQ) is a widely investigated CPP derived from the HIV-1 virus that can efficiently permeate through the cell membrane in various tissues [60]. Gold NPs decorated with TAT CPPs have been shown to increase in vivo brain accumulation by 4.8-fold in GBM-bearing mice [61]. Sharma et al. compared the BBB transport ability of three different CPPs, including TAT, penetratin, and mastoparan, by conjugating them onto Tf-functionalized liposomes and found that all three CPPs facilitated significant improvement in transport in a 3D brain tumour model compared to singly functionalized Tf or CPP liposomes [62]. Among the three CPPs, Tf-penetratin and Tf-TAT liposomes significantly enhanced brain penetration with 3.67% and 2.89% injected dose per gram of tissue in an in vitro brain tumor model, respectively [62]. Furthermore, TAT-conjugated mesoporous silica NPs (MSNs) delivering methotrexate (MTX) for glioblastoma treatment showed a significant 31.1-fold increase in brain accumulation in mice compared to free drugs, while unmodified MSNs only showed a 2.8-fold increase [63].

CPPs are uniquely positioned to facilitate the delivery of nucleic acid drugs like messenger RNA (mRNA), siRNA, and miRNA given their ability to cross the cell membrane into the cell cytosol, where these therapeutics can activate or inhibit gene expression of various proteins. Specifically, Srimaneea et al. developed a delivery system for siRNA to glioblastoma cells by modifying the LRP1-targeting peptide Ang to a hexaglutamate (E6) sequence at the N-terminus with a 6-aminohexanoic acid linker to yield a positive peptide TG1 that can non-covalently interact with the negatively-charged CPP PepFect14 (PF14) [64]. The resulting PF14:siRNA/TG1 NP complexes facilitated an 80% knockdown of luciferase expression in U87 glioma cells, a twofold increase from the CPP alone, suggesting the effectiveness of combining the cell-penetrating and brain and glioma targeting abilities of various peptides to form the optimal ligand [64].

### Limitations to CPP-functionalization

Despite promising results, various limitations related to CPP-functionalized NPs for brain delivery exist, including non-specific cell penetration and increased cytotoxicity. Since CPPs enhance penetration in all tissues and cell types via non-specific mechanisms, including adsorptive-mediated endocytosis, conjugating CPPs to NPs can increase the off-target effects in other organs or non-diseased cells. Strategies that have been proposed to overcome these limitations include CPP shielding with a targeting peptide [31], conjugating a targeting sequence to the CPP [65, 66], and incorporating a pH-activable CPP [33, 65].

To prevent non-specific cell penetration induced by the CPPs, Zhu et al. conjugated the RMT targeting peptide Ang to NPs via a long PEG linker (PEG6000) and the TAT CPP via a short PEG linker (PEG2000). Ang shields the TAT peptide during blood circulation, but when Ang binds to LRP-1 on the BBB and glioma cells, TAT is close enough to the cell membrane to induce penetration. This approach increased in vitro BBB transport by 1.8-fold and 4.2-fold and prolonged the median survival time of glioma-bearing mice by 9 days and 20 days compared to Ang-singly modified NPs and unmodified NPs, respectively [31].

Liu et al. designed a fusion peptide composed of a CPP octa-arginine (R8) conjugated to the retro-inverso isomer of RGD, called dGR, that retains the ability to target integrin αvβ3 receptors expressed on BCECs and glioma cells [66]. This sequence, called R8-dGR, also consists of an RXXR C-terminal motif that can specifically bind to neuropilin-1 (NRP-1) receptors expressed on BCECs and glioma cells, facilitating increased cellular uptake by the C-end rule [67]. When conjugated onto liposomes, the R8-dGR peptide showed superior transport across the BBB monolayer and cellular uptake in glioma cells in vitro and accumulation in the brain and glioma region in mice compared to R8-RGD (no NRP-1 targeting), R8-EGR (no integrin targeting), and R8 [66, 68].

To overcome poor penetration to deeper regions within the tumour parenchyma, Shi et al. designed a fusion peptide composed of cyclic RGD, a peptide that targets integrin αvβ3 overexpressed on BCECs and glioma cells, and TH, a pH-activable CPP that is slightly negative at normal physiological condition but becomes protonated into positive charge at pH ~ 6.5 near tumour cells [65]. Liposomes conjugated with this fusion peptide displayed deeper penetration into C6 spheroids and, when loaded with paclitaxel (PTX), significantly prolonged the median survival time of glioma-bearing mice to 45 days compared to 38.5 days and 27 days with cyclic RGD and TH functionalized liposomes, respectively.

## Designing peptides for brain-targeted nanoparticles

**Fig. 3:**
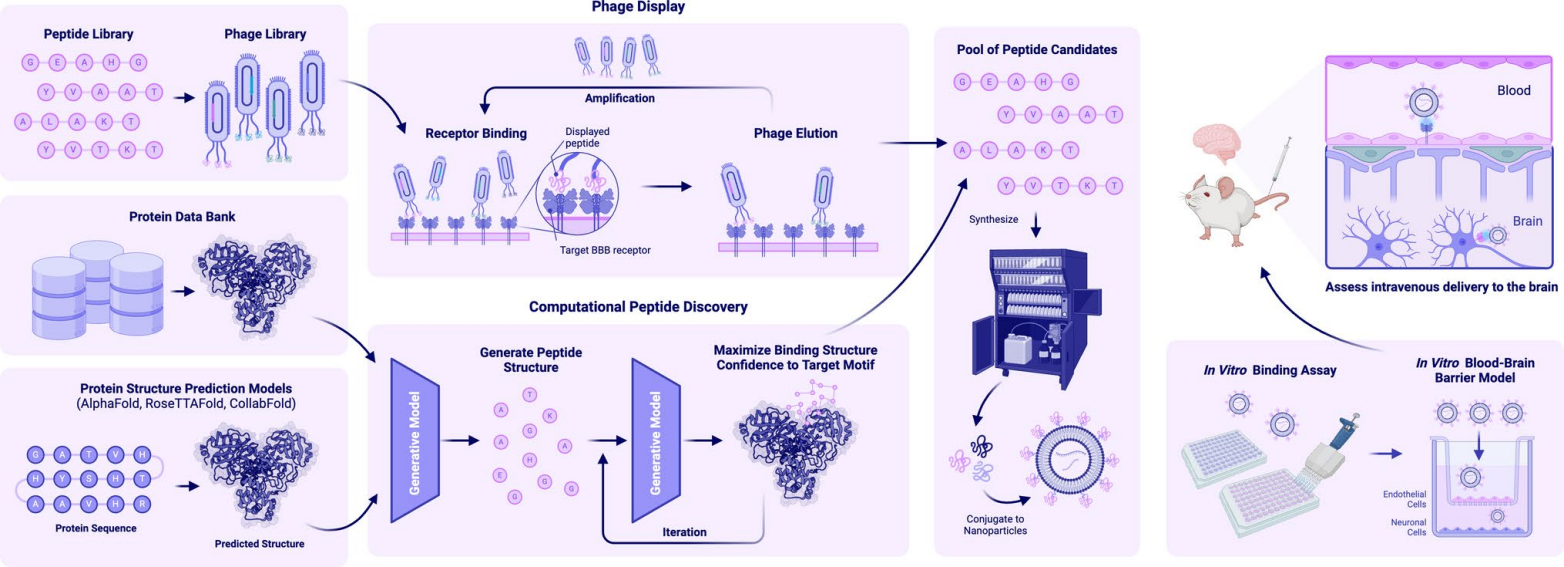
Pipeline for de novo discovery and validation of novel brain-targeting peptides for nanoparticle (NP) functionalization. First, phage display or generative deep-learning models are used to discover peptides with high affinity to the target receptor on the blood–brain barrier. Candidate peptides are synthesized and conjugated to NPs. Finally, peptide-functionalized NPs are validated in vitro and in vivo for delivery past the blood–brain barrier

Although existing peptides are promising BBB-targeting agents, there remain limitations to that can be solved through rational engineering. These include the lack of known peptide binders for various disease-homing and cell-type specific receptors, the susceptibility for protease-mediated degradation in the bloodstream, and the need for a robust targeting strategy for BBB-targeting and guidance to diseased cells. This section provides an overview of the key approaches to designing brain-targeted peptide-functionalized NPs. It begins with discussing phage display, a technique for identifying novel BBB-targeting and disease-homing peptides. We discuss the unique properties of fusion peptides that target multiple receptors, enabling a cascading BBB-crossing and disease-specific targeting strategy. The discussion then highlights the potential of cyclic peptides, which offer increased stability and binding specificity. Finally, additional considerations for peptide design are reviewed, including modifications to improve stability, targeting efficiency, and compatibility with NP platforms. These strategies exemplify the progress and promise of peptide engineering for overcoming the barriers of brain drug delivery (Fig. 3).

### Phage display

Phage display is a tool that enables screening libraries of up to 10^9^ peptide sequences to identify novel peptides that bind specifically to BCECs or disease-specific regions of the brain. A library of peptides is fused to the coat proteins on the surface of a bacteriophage, with each peptide displayed on the surface of a unique phage and introduced into the brain in situ. Then, after perfusion, only the phages with strong binding to the target tissue are recovered and sequenced to determine enriched peptides for further investigation [69].

To identify brain-targeting peptides, Rooy et al. leveraged a two-stage approach where a random library of 15-mer peptides was screened for enrichment in a perfused mouse brain, and the peptides with the highest enrichment were validated in vitro for binding affinity to human BCECs. Enrichment in the perfused lung and binding affinity to non-brain endothelial cells were investigated to evaluate binding specificity. This technique identified two peptides, GLA and GYR, with six- and fivefold enhanced brain targeting and 8.5- and 48-fold preference for the brain over the lung after intravenous injection in mice, respectively [70]. Furthermore, Wu et al. showed that GYR can be chemically modified to self-assemble into nanocarriers to deliver BACE1 siRNA across the BBB and transfect neurons and microglial cells, resulting in 50% downregulation after a single intravenous injection in mice [71].

To identify peptides that accumulate in sites of brain injury, Mann et al. injected a library of cyclic peptides of the form CX7K on a T7 phage following an inflicted brain injury in mice [72]. They found that a specific sequence CAQK was highly enriched in the region of the injury, comprising 22% of the recovered phage [72]. Conjugating the novel four-amino acid peptide CAQK to porous silicon NPs loaded with siRNA (PSiNPs) resulted in a 35-fold higher accumulation at the brain injury site compared to control peptide-NPs and 70% silencing after IV injection into GFP transgenic mice.

Since phages can cross a transcellular membrane via macropinocytosis, they are an effective tool for identifying peptides that exploit macropinocytosis to permeate the transcellular BBB membrane [73]. Given the enhanced target-binding affinity and stability of cyclic peptides, Yamaguchi et al. used phage screening to identify novel cyclic peptides from a library of 10^9^ peptides by inserting the peptide-covered phages into a BBB-transwell model and sequencing the phages to identify the peptides with the highest concentration in the abluminal side of the membrane [74]. After further experiments, a novel cyclic peptide (SLS) identified during phage display was shown to facilitate enhanced BBB-permeability in vitro and after intravenous injection in mice when conjugated to liposomes [74].

### Fusion peptides

To efficiently deliver therapeutics for neurological disorders, various studies have focused on a dual-stage targeting approach, functionalizing NPs with ligands that first facilitate crossing of the BBB before guiding the NPs to specific cell types and neuronal tissue involved in disease. This strategy prompted the design of fusion peptides that combine two peptides with distinct target proteins, demonstrating enhanced targeting properties over functionalizing the NP surface with multiple distinct peptides (Fig. 2) [75, 76].

Guo et al. combined the established BBB-penetrating peptide TGN and a neuron-binding peptide Tet1 with a four-glycine linker to form a novel fusion peptide TPL, which showed superior BBB- and neuronal-targeting ability than either single peptide modification or dual peptide modification on PEG-PLA NPs, suggesting that fusion peptides enhance the synergistic mechanisms of its constituent peptide components [75]. Furthermore, TPL degraded much slower than either mono-ligand peptides (17% compared to 50% after 2 h), which can likely be attributed to its higher isoelectric point causing stronger repulsion between the NP and the physiological environment, demonstrating the potential to modulate the properties of peptides via fusion. In an AD mouse model, the TPL-NPs loaded with fluorescent dye DiR led to the largest increase in delivery to the brain following intravenous injection, demonstrating 5.68-fold higher fluorescence compared to untargeted NPs, 3.46-fold higher compared to NPs singly targeted with CGN peptide, and 1.73-fold higher compared to dual-targeted NPs with CGN and Tet1. Given that CGN has a higher BBB-targeting ability than TGN present in the fusion peptide, this suggests that the enhanced BBB-targeting capability of TPL is a result of the unique properties introduced during the fusion process. Furthermore, TPL demonstrated decreased off-target accumulation in the liver and a significant increase in neuron-specificity, with 92.2% accumulation near neurons and an 8.73-fold and 2.41-fold higher accumulation compared to CGN-functionalized and CGN and Tet1 dual-functionalized NPs respectively.

Furthermore, fusion peptides have been developed specifically for dual targeting of the blood-tumour barrier (BTB) and glioma cells. Zhang et al. combined a 7-amino acid peptide GICP (glioma-initiating cell peptide) discovered via phage display to target the VAV3 protein overexpressed on glioma cells with the D-form A7R peptide that targets both vascular endothelial growth factor receptor 2 (VEGFR2) and neuropilin-1 (NRP-1) abundant on blood vessels on the BTB to form a novel Y-shaped fusion peptide that is capable of cross the BTB and target glioma cells [76]. The fusion peptide showed enhanced resistance to degradation, increasing circulation time from 4 h for single GIPC peptides to 12 h for the fusion D-A7R-GIPC peptides. Furthermore, they exhibited increased uptake in U87MG and HUVEC cells in vitro and increased accumulation in the tumour region in mice bearing a subcutaneous U87MG tumour compared to A7R or GIPC alone, likely owing to the affinity to complementary binding receptors on both the BTB and glioma cells [76].

Fusion peptides also provide various levers for rational design that improve therapeutic properties, including linker design and desirable conformational changes. As long linker lengths can result in increased protease degradation and short linker lengths can inhibit necessary conformational changes of either peptide component following receptor binding, it is crucial to determine the optimal linker length for fusion. By comparing fusion peptides with no linkage, two-, four- and six-glycine linkers, Guo et al. demonstrated that the four-glycine linker fusion peptide showed superior affinity toward BCECs and the GT1b receptor than other linker lengths and single-ligand peptide components [75]. The linker design can also enhance the hydrophobicity of the fusion peptide, resulting in increased cellular uptake [76]. In addition, the degree of conformation change upon interacting with a target receptor is a determinant of binding affinity [77]. The fusion peptide TPL showed greater conformational change upon interacting with the GT1b receptor compared to the single peptides, transforming from random coils to α-helices, indicating that fusion can enable structural conformations needed to bind to the target receptor. Future studies should systematically investigate the effects of varying the linker length and composition on the targeting properties. These studies could involve generating libraries of different linkers using programs like LINKER [78] or generative computational models that are conditioned to generate linkers that retain target-binding affinity [79].

### Cyclic peptides

Cyclic peptides are of increasing therapeutic interest due to their favourable properties, including high binding affinity and specificity, stable structure, membrane permeability and low degradation (Fig. 2) [80]. Due to the cyclic connection, cyclic peptides have higher structural stability, which restricts the number of possible conformations, increasing the probability of the peptide occurring in the target-binding conformation and decreasing the likelihood of off-target binding conformations as seen in X-ray structures of protein-peptide complexes [81]. The stable conformations also prevent cyclic peptides from binding to proteases, reducing their susceptibility to degradation in the bloodstream [80]. Cyclization also allows peptides to form intramolecular hydrogen bonds, desolvating the peptide and enabling it to pass the hydrophobic lipid bilayer [82]. Given their favourable properties, several studies have conjugated brain-targeted cyclic peptides to the surface of NPs [65, 68, 72, 83–85].

One of the most extensively explored cyclic peptides for neurotherapeutic delivery is cRGD (cyclic Arg-Gly-Asp), which targets α_v_β_3_ and α_v_β_5_ integrins that are abundant on the endothelial cells of tumour blood vessels and glioblastoma (GBM) cells [65, 83, 85, 86]. After intravenous injection into an orthotopic mouse model of U87MG human GBM, Miura et al. found that cRGD-conjugated polymeric micelles can enhance penetration into the tumour vasculature via integrin-mediated active transport pathways [85]. In addition, conjugating cRGD onto PEG-PLA micelles and liposomes loaded with PTX increased survival time by over 10 days in tumour-bearing mice compared to the free drug and over 7 days compared to untargeted NPs, which increased further when co-functionalized with the TR cell-penetrating peptide [65, 83]. To overcome the BBB and the blood–brain tumour barrier (BBTB) for glioma therapy, Chen et al. co-functionalized liposomes with cRGD and Peptide-22, another BBB-targeting peptide, demonstrating the greatest uptake in the glioma in mice and extending survival times by over 3 days compared to either single-functionalized liposomes [86]. This suggests a synergistic effect of targeting both LDLR and α_v_β_5_ integrins on the BBB/BBTB surface.

Other brain-targeting cyclic peptides have also been discovered for the treatment of neurological disorders. After screening a library of 42 peptides for binding affinity to LRP1, Sakamoto et al. discovered a novel cyclic peptide KS-487 containing a cyclic disulphide linkage that selectively binds to the cluster IV domain of LRP1 abundant on the BBB, with higher BBB-permeability than the linear peptide Angiopep-2 [87, 88]. By conjugating KS-487 to micelles or liposomes loaded with the KS-133 peptide, a therapeutic peptide that inhibits VIPR2 involved in schizophrenia, they showed that the KS-487-targeted NPs improved cognitive dysfunction in a schizophrenia mice model [87, 89].

Cyclizations can be introduced into linear peptides via various chemical reactions, including side-chain cyclization commonly involving disulphide reactions between cysteine residues and head-to-tail cyclization, which forms an amide bond between the amino and carboxylic acid groups of two non-adjacent residues [90]. Given the success of cyclic peptides in facilitating targeted NP-delivery, future studies should focus on introducing cyclizations to a wider range of existing brain-targeting peptides and comparing the brain-targeting properties to their linear counterparts in addition to designing novel cyclic peptides through phage display or computational design methods that specifically target diverse receptors involved in neurological disorders.

### Additional design considerations

The ability to fine-tune peptide structures through chemical modifications, such as cyclization, backbone alterations, and sidechain functionalization, has revolutionized their application in drug delivery systems. Even minor structural modifications can impact a peptide’s stability, target specificity, and functional properties. Among these strategies, the stereoisomeric configuration of peptides, the incorporation of acid-cleavable linkers, and the rational design of peptide conjugates have emerged as transformative approaches to overcoming the challenges of delivering therapeutics to the brain.

D- and L-form peptides consist of stereoisomeric amino acids that differ only in the location of the amino group on the alpha carbon (Fig. 2). Studies have shown that retro-inverso (RI) D-form peptides have higher stability and are less susceptible to proteolysis by enzymes in the bloodstream than their L-form counterparts while retaining their target-binding affinity [91]. Wei et al. compared the stability and BBB targeting of Ang, an l-peptide, with its retro-inverso (RI) d-peptide isomer conjugated with micelles. Although the RI peptide showed lower cellular uptake in BCECs in vitro, it showed nearly no degradation in blood after 8 h, whereas 85% of the L-form peptide degraded within 2 h, resulting in an overall higher micelle distribution in the brains of mice in vivo [34]. Another study showed that the RI analog of a 16-amino acid peptide from antennapedia has a 3.4-fold greater concentration after incubation with cultured neurons by replacing L-form RR and KK motifs with high affinity to neuronal proteases with their D-form counterparts resistant to degradation [92]. In addition to improving stability, reversing the chirality of targeting peptides has also been used as a strategy when designing fusion peptides to ensure that the critical residues involved in target binding are exposed at the terminus after fusion [76].

Peptides can also be easily conjugated to acid-cleavable moieties to facilitate deep penetration into the brain parenchyma and reduce shielding between peptides. Cai et al. designed a dual-functionalized NP with acid-cleavable T7 peptides and neuron-targeting Tet1 peptides to co-deliver BACE1 siRNA and D-peptide for AD treatment [25]. After T7 facilitates TfR-mediated endocytosis into BBB endothelial cells, the change in the acidic environment detaches the NP from the T7 peptides via the acid-cleavable PEG linker, releasing the NPs from the receptors and allowing them to escape from the endo/lysosomes. Upon entering the brain parenchyma, the NPs accumulate on target neurons via the Tet1 peptide targeting. This delivery platform significantly reduced amyloid plaques in the brains of AD mice and BACE1 gene expression in target cells in vivo compared to mono-functionalized NPs. The acid-cleavable group also prevents shielding of the Tet1 peptide by T7 after crossing the BBB, ensuring that Tet1 can easily bind to their receptor targets on neuronal cells. Prior works have also used acid-cleavable linkers for targeting other ligands, such as proteins. Gold NPs decorated with endogenous Tf protein with an acid-cleavable diamino ketal (DAK) linker showed a 2.7-fold increase in brain penetration in mice compared to non-cleavable Tf-AuNPs by enabling the release of bound AuNPs into the brain parenchyma upon transcytosis into lower pH [93]. Although DAK is highly stable at pH 7.4 and can easily be introduced as a linkage to NPs, they suffer from slow cleavability. Therefore, future work should focus on designing linkers with high compatibility with NP-peptide conjugation in addition to rapid cleavability during transcytosis.

When designing peptide-targeted NPs, it is crucial to consider how the protein corona (PC) affects targeting efficacy. After intravenous injection, proteins in the bloodstream can adsorb onto the surface of NPs, forming a PC [94]. Although studies have found that the formation of a PC has attenuated the binding affinity of targeted NPs, other studies have leveraged peptides to control the specific protein populations in the PC to enable brain-targeting capabilities [94]. Specifically, Zhang et al. conjugated amyloid β‐protein (Aβ)‐CN peptide to nano micelles, which enables specific binding to the lipid-binding domain of ApoE in the bloodstream and facilitates ApoE-mediated targeting of LDLR and LRP1 on the BBB and glioma cells via the receptor-binding domain [95]. This approach significantly prolonged median survival times in glioma-bearing mice from 28 days for non-functionalized nano micelles to 45 days for functionalized nano micelles [95]. Without encouraging the formation of a specific PC, its formation can also be mitigated by tuning the charge, hydrophobicity, and linker length of targeted NPs. The formation of the PC on NPs in fetal bovine serum showed that anionic NPs and hydrophobic NPs adsorbed less diversity and mass of proteins compared to cationic NPs, suggesting that the conjugation of peptides with anionic or hydrophobic properties could reduce the formation of the PC [96]. In addition, it was shown that conjugating cyclic RGD peptide with longer PEG linkers increased the adsorption of proteins on gold NPs [97]. In total, future studies should investigate the design of novel PC-modulating peptides that encourage the formation of a favourable brain-targeting PC or cyclic peptides with a highly stable conformation to prevent binding with off-target and endogenous proteins.

## Computationally designed peptides for brain targeting

The emergence of generative models for de novo protein and peptide design holds the potential to accelerate the design of novel brain-targeting peptides with enhanced properties. For peptides containing only the 20 canonical amino acids, the space of all possible peptides is 20^*L*^, where *L* is the length of the peptide sequence. If chemically modified and non-canonical amino acids are considered, the space of possible sequences is even larger. Current rational design strategies rely on taking fragments of existing naturally binding proteins, editing existing peptide binders to enhance specific properties, or combining multiple peptides with known mechanisms, limiting the potential protein receptor targets to only those with known or peptide binders. This leaves out the vast set of surface receptors without known peptide binders.

Traditional methods of peptide discovery remain a bottleneck, as they often involve screening billions of libraries of random peptide sequences for high target affinity [98, 99]. Furthermore, generating libraries for protein receptor targets with no known binders or with no experimentally determined structure is an arbitrary process which fails to narrow down peptides with promising properties for further experimental validation. Generative deep-learning models overcome these challenges by learning key motifs across large databases of experimentally validated peptides to efficiently sample diverse sequences within the subspace of peptides that resemble chemically feasible peptides (Fig. 3).

### Generative models for de novo peptide discovery

Generative protein and peptide design can be broadly categorized into sequence-based and structure-based models depending on the type of representation that the model is trained to generate [100]. Sequence-based models are trained solely on sequence representations of peptides like amino acids or Simplified Molecular Input Line Entry System (SMILES) strings [101] from large databases, where each sequence is split into tokens that act as a vocabulary for all peptide sequences. Since the structural conformation and chemical properties of peptides are largely determined by their distinct sequence of amino acids, sequence-based models are trained to effectively capture these diverse properties in numerical feature embeddings, where the embedding is a vector that captures the unique properties of each token. These embeddings capture local and global features of the peptide — including hydrophobicity, charge, bonding interactions, and pH — as well as the context in which each token appears across the database. Then, these embeddings are leveraged for de novo peptide design by training a generative model that produces new combinations of peptide token embeddings from the learned distribution of feasible combinations and decoding them into the corresponding amino acid or SMILES sequence. Although various architectures have been explored for modelling sequences, the most promising are Transformer-based models which take a weighted sum of the feature embeddings from the global peptide context from each token in the sequence to capture the long-range relationships between residues [102]. ESM (Evolutionary-Scale Modelling) [103] is a family of Transformer-based models trained on 65 million amino-acid sequences from the UniProt [104] protein database. ESM is trained by masking a subset of tokens in the training sequences and minimizing the error associated with reconstructing the original sequence from the parameterized feature embeddings, such that the final model can generate embeddings from unseen protein sequences that capture protein structure and properties. The pre-trained ESM backbone has been used in various generative models for peptide design and discovery, specifically masked diffusion models that learn to reconstruct a peptide sequence from a sequence of unknown mask tokens [105–108].

Structure-based models are trained on the secondary structure of peptides or peptide-protein conformations to generate novel peptide structures, which can be further decoded into a sequence for synthesis. These models are explicitly trained on peptide structure, allowing them to generate peptides given specific structural constraints, such as a target protein binding site [109]. However, a major limitation of structure-based models is an efficient conversion from the structural representation to a sequence representation accurately forms the generated structure. To overcome this problem, a class of deep learning models called inverse folding models have been developed that learn to predict a protein or peptide sequence from its three-dimensional representation [110, 111]. Since peptides have a shorter sequence length than the proteins used to train inverse folding models and typically form only secondary structures rather than tertiary structures like proteins [112], the structural data for peptides is sparse, and conversion from structure to sequence can result in sequences that do not exhibit the desired properties. Therefore, sequence-based models are better suited for generative peptide design as opposed to structure-based models given their ability to capture the inherent properties within the peptide sequence from larger datasets while bypassing the inaccuracies introduced in the conversion from sequence to structure.

### Objectives for BBB-targeted peptide discovery

A most common objective for generative peptide discovery is target-aware generation, where the generation process is conditioned on the structure or sequence of a binding motif or full sequence of a target protein. Target-aware models are trained on peptide-protein pairs with known binding affinity or structure to learn different features that determine binding, like hydrophobicity, covalent and non-covalent interactions, structural complementarity, among others. This enables the model to generate novel peptide binders that encapsulate the interaction features involved with high binding affinities given the amino acid in the protein target, even if the specific protein has not been trained on. Target-aware models can also be classified as sequence-based or structure-based, depending on the input representation of the target protein. Target-sequence-based models take an amino acid sequence of the protein target or binding motif from databases such as UniProt [104], convert the sequence into a sequence of feature-rich embeddings, and generate a complementary peptide binder sequence that has high affinity given the chemical properties and structural features represented in the protein embeddings [108, 113]. Target-structure-based models take the three-dimensional structure of the target protein or a specific binding pocket to generate a novel peptide sequence or structure that complements the target structure [114, 115]. Protein structures can be obtained from resources like the Protein Data Bank (PDB) for receptors with known structures. In cases where the receptor structure is unavailable, alternative approaches include leveraging sequence-based generative models or employing structure-prediction tools to infer the receptor’s conformation [116–118]. While both methods enable conditioning on target binding affinity, sequence-based approaches expand the space of potential targets to proteins without experimentally validated or stable tertiary structures by relying solely on the intrinsic features and context encoded in the amino acid feature embeddings.

Property-conditioned design is an alternative objective that can enable the design of BBB-targeting peptides. Instead of conditioning on a specific target protein or receptor, the model is conditioned to generate peptides with a specified property, like BBB permeability. These models leverage classification models that, given a peptide sequence, can predict a score that determines how well the sequence performs in the given objective. The classification models are trained on labelled, experimental data to learn the features that differentiate a sequence with a given property from those without. Several machine learning models have been developed to predict the permeability of molecules to cross the BBB [119–122]. Integrating these classifiers with score-based generative models can reward peptides with high scores and guide the model to generate peptides with enhanced properties. This framework has been explored for the discovery of BBB-permeable small-molecule drugs using reinforcement learning [123] and multi-property conditioned BBB-targeted peptide generation, where the peptides are conditioned on not only target binding affinity but also membrane permeability to enable cellular transfection of therapies with intracellular mechanisms of action, solubility for increased drug loading, and hemolysis and non-fouling to minimize off-target effects using a set of trained property classifiers on labelled peptide datasets [124].

### In vitro validated tools for computational peptide design

This section reviews in vitro validated generative deep-learning models for generating libraries of brain-targeting peptide candidates tailored for drug delivery applications. These strategies provide a foundation for designing peptides with high specificity and therapeutic potential, addressing critical challenges in targeted drug delivery (Table 2).

**Table 2:** Computational models for peptide discovery and design with in vitro results

Model	Input Format	Model Architecture	Potential Application for BBB Targeting	Citation
PepPrClip	Amino acid sequence of target protein	Generate embeddings that reflect the similarity of protein-peptide pairs; sampling new embeddings from Gaussian distributions around the natural peptide embeddings; converting from embedding to sequence using a decoder	Designing peptides that bind to a target receptor. Useful for protein targets with no known structure and low confidence predicted structures	[107]
PepMLM	Amino acid sequence of target protein and specified length of peptide binder	Masked discrete diffusion language model that iteratively unmasks the peptide binder sequence given the input protein target sequence	Designing peptides that bind to any site of a target receptor. Useful for protein targets with no known structure and low confidence predicted structures	[125]
RFpeptides	PDB structure file of target protein	Diffusion-based model that generates the backbone coordinates of a peptide; message-passing neural network to convert backbone structure into peptide sequence	Cyclic peptide design with enhanced properties over linear peptides including high binding affinity, stable structure, membrane permeability, and low enzymatic degradation	[114]

For sequence-only generation, Peptide Prioritization via CLIP (PepPrCLIP) [126] introduced a generative peptide design framework that applies Gaussian noise to the feature-rich embedding representations of natural peptides generated by ESM-2 [103], sample embeddings from the latent space near the natural peptides, and decodes them into novel peptide sequences. Since ESM-2 is trained on millions of naturally occurring proteins and peptides, it can capture the biological properties of peptides such that two peptides with similar properties are mapped to vector embeddings with high similarity. After sampling and decoding, the de novo sequences are fed into a contrastive language-image pretraining (CLIP) [127] classifier that generates a score estimating the binding affinity to a target protein. After generating only 20 peptide sequences targeted to the enzyme UltraID not present in the training set, four candidates successfully inhibited the enzyme’s activity by over 75% in human embryonic kidney (HEK) 293 T cells, demonstrating the model’s capability of narrowing down the search space of high-affinity binders to unseen targets. Furthermore, from a pool of six generated peptides with high predicted affinity to β-catenin, two of the candidates successfully guided ubiquibodies (uAbs) —genetically encodable protein-degrading E3 ubiquitin ligases—to β-catenin in colorectal adenocarcinoma DLD1 cells, resulting in 50% greater degradation compared to the non-guided control. In addition to structurally stable target proteins, PepPrCLIP-generated peptides could bind to the highly disordered oncogenic fusion protein SS18-SSX, reducing expression levels by > 40% in vitro.

PepMLM [125] is another sequence-based model that learns to generate target-specific peptide binders by masking the full peptide sequence and concatenating the masked sequence to the end of its target protein sequence from known protein-peptide pairs. The model is trained to iteratively reconstruct the peptide given the embedding representations of the target protein and previous amino acids. After training, the model can generate de novo peptide sequences given a protein sequence by following a similar unmasking strategy. Three of five selected peptides generated for the aggregation-prone mutant huntingtin protein (mHTT) were able to guide protein degraders to the target, resulting in significant and complete degradation of mHTT. Altogether, sequence-based generative peptide design is capable of generating de novo peptide sequences for virtually any protein target, including both structurally stable and disordered proteins, which not only significantly cuts the cost and time associated with traditional screening methods but can bias the search process towards diverse peptides with optimal binding properties. The promising in vitro results from these studies can be extended towards accelerating the design of peptides that target surface receptors on the BBB and diverse brain cell types, facilitating active targeting of cells with no known binding ligands.

Several protein-structure prediction models exist to incorporate the target-protein structure into the peptide design process for receptors without experimentally validated structures, including AlphaFold [128], RoseTTAFold [116], and ColabFold [117]. These models predict unseen protein structures from the multiple-sequence alignment (MSA) containing protein homologs with known structure. The output of these models include a PDB file containing the structural coordinates of the target protein and several confidence metrics, including the pLDDT score that measures per-residue prediction confidence between 0 to 100, where scores > 70 indicating accurate backbone prediction and scores > 90 accurate backbone and sidechain predictions. Target protein with no experimentally validated structure but high confidence structure predictions can be used as input to structure-based or hybrid generative peptide models that incorporate both structure and sequence.

Given the high binding affinity and specificity, stable structure, membrane permeability, and low degradation of cyclic peptides [80], there have been major advancements in deep learning models tailored for cyclic peptide representation and design. RFpeptides [114] is a target-structure based diffusion model built on the highly successful RFdiffusion protein-generation model [109] specifically finetuned to generate cyclic peptides where the first and last amino acids can form a chemical bond. RFpeptide has been shown to generate high-affinity binders to protein targets with no known binders and no experimentally determined structure, a feat that is infeasible for library-based approaches. To design peptide binders to Rhombotarget A (RbtA), a protein with no known structure, Rettie et al. predicted the 3D structure of the RbtA using RoseTTAFold2 [116], generated 20,000 peptide structural backbones using RFdiffusion [109], and finally extracted four amino acid sequences given each peptide backbone using ProteinMPNN [110]. The strongest binder among 26 tested in vitro showed a high dissociation constant of 9.4 nM. RFpeptides also generated various binders to structurally diverse binding sites, including myeloid cell leukemia-1 (MCL1), MDM2 involved in tumour growth and survival, and Gamma-aminobutyric acid type A receptor-associated protein (GABARAP). Given these results, RFpeptides can be a powerful tool to generate cyclic peptides with enhanced properties compared to linear peptides [80] that selectively target a diverse array of receptors without known structure or binders on the BBB.

### Directions for computationally designed peptides

Although nAchR, TfR, and LDLR are highly expressed in the brain and have enhanced the delivery of various peptide-conjugated NPs across the BBB, they are also expressed on off-target organs [50]. Specifically, TfR is also highly expressed in the spleen and LDLR in the liver, spleen, and lungs. This may result in off-target effects and higher effective dosages. In addition, many cell types in the brain that are involved in neurological diseases, including astrocytes, microglia, and cells in diseased states, have no known peptide binders. Therefore, computational peptide design strategies have great potential in generating de novo peptide binders to receptors specifically expressed in the brain or specific subpopulations of brain cells. The family of super-conserved receptors on the brain (SREB), including G protein-coupled receptors GPR27 (SREB1), GPR85 (SREB2), and GPR173 (SREB3), are potential targets with increased brain specificity as they are almost exclusively expressed in the CNS [118]. This family of receptors have been shown to modulate various neurological and psychiatric diseases, including schizophrenia [129, 130] and autism spectrum disorder [131]. Chen et al. found that SREB2 has a strong inhibitory effect on the differentiation and survival of new neurons, leading to adult neurogenesis in the hippocampus in a schizophrenia mouse model [129]. However, no endogenous ligands for SREB1 and SREB2 are known and only two less characterized ligands are known for SREB3 [129, 132–134]. The SREB family are only a few examples of receptors with no known ligands expressed in the brain, which could act as targeting proteins for the treatment of many untreatable neurological disorders, such as schizophrenia and other psychiatric disorders. This motivates future studies that employ computational techniques to generate candidate peptide binders with high affinity towards these receptors for further in vitro and in vivo investigations.

## Conclusions

Peptides have emerged as a class of targeting ligands to enhance therapeutic delivery, presenting several advantages including ease of synthesis, small size, reduced cost, and lower immunogenicity over other classes of ligands like proteins and antibodies. In parallel, the rational design of NP-based delivery vehicles for intravenous delivery of various drugs has shown significant promise. Next-generation nucleic acid therapeutics in particular require NP encapsulation, as seen with the recent FDA-approval of Onpattro, a siRNA therapy for polyneuropathy, and the mRNA COVID-19 vaccines, both of which are encapsulated by lipid nanoparticles (LNPs) [135]. Despite the biological barriers that exist for intravenous delivery of drugs to the brain, the combination of target-specific peptides conjugated on optimized NP systems has been shown to synergistically facilitate low cytotoxicity, low degradation, BBB-targeting, and cellular uptake of various drug payloads for a wide range of neurological disorders, including glioma, AD, PD, stroke, among others. Specifically, RVG29, T7, Angiopep-2, and mApoE peptides, which target receptors nAchR, TfR, and LDL receptors widely expressed on the BBB and brain cell types, have shown promise in neurological disease models. These peptides also serve multiple targeting functions due to the upregulation of TfR and LDL receptors on glioma cells and nAchR on neurons. Rational design strategies involving these well-established peptides have been developed to enhance the brain-targeting properties — including fusion of multi-target binding peptides, cyclization, and co-functionalizing with cell-penetrating peptides.

Since rational design strategies limit the potential targets of peptide-functionalized NPs to only those with known peptide binders, there is a demand for de novo peptide discovery. To this end, traditional techniques like phage display and emerging advancements like generative deep learning must be leveraged to widen the range of therapeutic pathways, with a specific focus on achieving brain-specificity and homing to cells involved in neurological disease.

Despite the significant advancements of brain-targeted NPs, there remain challenges to bringing these platforms to the clinic [136]. In particular, ensuring that the targeting peptides satisfy the range of properties necessary for therapeutic viability, including half-life, non-fouling, and non-hemolysis, is imperative for success in later clinical stages. This requires rigorous experiments validating the properties of candidate peptides in vitro and in vivo, a process which can be accelerated by narrowing down candidates with the help of computational property predictors. Furthermore, scaling the development pipelines of brain-targeted NPs will require further optimization of high-throughput peptide synthesis [137], conjugation, and purification methods.

Through the work described in this review, we demonstrate that by bridging the gap between advances in computational design strategies and experimental research, we can design and discover potent peptide binders to overcome the greatest barriers to effective drug delivery to the brain, ultimately facilitating the next generation of CNS-targeted precision therapeutics.

## Data Availability

No data were generated in the preparation of this manuscript.

## References

[1] Teixeira MI, Lopes CM, Amaral MH, Costa PC. Surface-modified lipid nanocarriers for crossing the blood-brain barrier (BBB): A current overview of active targeting in brain diseases. Colloids Surf B Biointerfaces. 2023;221:112999. 10.1016/j.colsurfb.2022.112999. 36368148 10.1016/j.colsurfb.2022.112999

[2] Mitchell MJ, Billingsley MM, Haley RM, Wechsler ME, Peppas NA, Langer R. Engineering precision nanoparticles for drug delivery. Nat Rev Drug Discov. 2021;20(2):101–24. 10.1038/s41573-020-0090-8. 33277608 10.1038/s41573-020-0090-8 PMC7717100

[3] Kou L, Bhutia YD, Yao Q, He Z, Sun J, Ganapathy V. Transporter-Guided Delivery of Nanoparticles to Improve Drug Permeation across Cellular Barriers and Drug Exposure to Selective Cell Types. Front. Pharmacol. 2018;9, 10.3389/fphar.2018.00027. 10.3389/fphar.2018.00027 PMC5791163 29434548

[4] Blanco E, Shen H, Ferrari M. Principles of nanoparticle design for overcoming biological barriers to drug delivery. Nat Biotechnol. 2015;33(9):941–51. 10.1038/nbt.3330. 26348965 10.1038/nbt.3330 PMC4978509

[5] Patra JK, et al. Nano based drug delivery systems: recent developments and future prospects. J Nanobiotechnology. 2018;16(1):71. 10.1186/s12951-018-0392-8. 30231877 10.1186/s12951-018-0392-8 PMC6145203

[6] Pardridge WM. The blood-brain barrier: bottleneck in brain drug development. NeuroRx J Am Soc Exp Neurother. 2005;2(1):3–14. 10.1602/neurorx.2.1.3. 10.1602/neurorx.2.1.3 PMC539316 15717053

[7] Li J, et al. Development of Novel Therapeutics Targeting the Blood-Brain Barrier: From Barrier to Carrier. Adv Sci. 2021;8(16):2101090. 10.1002/advs.202101090. 10.1002/advs.202101090 PMC8373165 34085418

[8] Uchida Y, et al. Quantitative targeted absolute proteomics of human blood-brain barrier transporters and receptors. J Neurochem. 2011;117(2):333–45. 10.1111/j.1471-4159.2011.07208.x. 21291474 10.1111/j.1471-4159.2011.07208.x

[9] Gao J, Judy Xia Z, Gunasekar S, Jiang C, Karp JM, Joshi N. Precision drug delivery to the central nervous system using engineered nanoparticles. Nat Rev Mater. 2024;9(8):567–88. 10.1038/s41578-024-00695-w.

[10] Smith SA, Selby LI, Johnston APR, Such GK. The Endosomal Escape of Nanoparticles: Toward More Efficient Cellular Delivery. Bioconjug Chem. 2019;30(2):263–72. 10.1021/acs.bioconjchem.8b00732. 30452233 10.1021/acs.bioconjchem.8b00732

[11] Foroozandeh P, Aziz AA. Insight into Cellular Uptake and Intracellular Trafficking of Nanoparticles. Nanoscale Res Lett. 2018;13(1):339. 10.1186/s11671-018-2728-6. 30361809 10.1186/s11671-018-2728-6 PMC6202307

[12] Behzadi S, et al. Cellular uptake of nanoparticles: journey inside the cell. Chem Soc Rev. 2017;46(14):4218–44. 10.1039/C6CS00636A. 28585944 10.1039/c6cs00636a PMC5593313

[13] Liu Y, et al. Brain-targeting gene delivery and cellular internalization mechanisms for modified rabies virus glycoprotein RVG29 nanoparticles. Biomaterials. 2009;30(25):4195–202. 10.1016/j.biomaterials.2009.02.051. 19467700 10.1016/j.biomaterials.2009.02.051

[14] Arora S, Layek B, Singh J. Design and Validation of Liposomal ApoE2 Gene Delivery System to Evade Blood-Brain Barrier for Effective Treatment of Alzheimer’s Disease. Mol Pharm. 2021;18(2):714–25. 10.1021/acs.molpharmaceut.0c00461. 32787268 10.1021/acs.molpharmaceut.0c00461 PMC10292003

[15] Qu M, et al. A brain targeting functionalized liposomes of the dopamine derivative N -3,4-bis(pivaloyloxy)-dopamine for treatment of Parkinson’s disease. J Controlled Release. 2018;277:173–82. 10.1016/j.jconrel.2018.03.019. 10.1016/j.jconrel.2018.03.019 29588159

[16] Gan L, Li Z, Lv Q, Huang W. Rabies virus glycoprotein (RVG29)-linked microRNA-124-loaded polymeric nanoparticles inhibit neuroinflammation in a Parkinson’s disease model. Int J Pharm. 2019;567:118449. 10.1016/j.ijpharm.2019.118449. 31226473 10.1016/j.ijpharm.2019.118449

[17] Hua H, et al. RVG29-modified docetaxel-loaded nanoparticles for brain-targeted glioma therapy. Int J Pharm. 2018;543(1–2):179–89. 10.1016/j.ijpharm.2018.03.028. 29555442 10.1016/j.ijpharm.2018.03.028

[18] Han Y, et al. Macrophage membrane-coated nanocarriers Co-Modified by RVG29 and TPP improve brain neuronal mitochondria-targeting and therapeutic efficacy in Alzheimer’s disease mice. Bioact Mater. 2021;6(2):529–42. 10.1016/j.bioactmat.2020.08.017. 32995678 10.1016/j.bioactmat.2020.08.017 PMC7492821

[19] Alvarez-Erviti L, Seow Y, Yin H, Betts C, Lakhal S, Wood MJA. Delivery of siRNA to the mouse brain by systemic injection of targeted exosomes. Nat Biotechnol. 2011;29(4):341–5. 10.1038/nbt.1807. 21423189 10.1038/nbt.1807

[20] Han EL, et al. Peptide-Functionalized Lipid Nanoparticles for Targeted Systemic mRNA Delivery to the Brain. Nano Lett. 2025;25(2):800–10. 10.1021/acs.nanolett.4c05186. 39688915 10.1021/acs.nanolett.4c05186

[21] Kuang Y, et al. T7 peptide-functionalized nanoparticles utilizing RNA interference for glioma dual targeting. Int J Pharm. 2013;454(1):11–20. 10.1016/j.ijpharm.2013.07.019. 23867728 10.1016/j.ijpharm.2013.07.019

[22] Yu M, et al. D-T7 Peptide-Modified PEGylated Bilirubin Nanoparticles Loaded with Cediranib and Paclitaxel for Antiangiogenesis and Chemotherapy of Glioma. ACS Appl Mater Interfaces. 2019;11(1):176–86. 10.1021/acsami.8b16219. 30525386 10.1021/acsami.8b16219

[23] Kim G, Kim M, Lee Y, Byun JW, Hwang DW, Lee M. Systemic delivery of microRNA-21 antisense oligonucleotides to the brain using T7-peptide decorated exosomes. J Controlled Release. 2020;317:273–81. 10.1016/j.jconrel.2019.11.009. 10.1016/j.jconrel.2019.11.009 31730913

[24] Bi Y, et al. T7 Peptide-Functionalized PEG-PLGA Micelles Loaded with Carmustine for Targeting Therapy of Glioma. ACS Appl Mater Interfaces. 2016;8(41):27465–73. 10.1021/acsami.6b05572. 27466824 10.1021/acsami.6b05572

[25] Cai L, et al. Endo/Lysosome-Escapable Delivery Depot for Improving BBB Transcytosis and Neuron Targeted Therapy of Alzheimer’s Disease. Adv Funct Mater. 2020;30(27):1909999. 10.1002/adfm.201909999.

[26] Zhao Y, et al. Dual targeted nanocarrier for brain ischemic stroke treatment. J Controlled Release. 2016;233:64–71. 10.1016/j.jconrel.2016.04.038. 10.1016/j.jconrel.2016.04.038 27142584

[27] Liang M, et al. Enhanced blood–brain barrier penetration and glioma therapy mediated by T7 peptide-modified low-density lipoprotein particles. Drug Deliv. 2018;25(1):1652–63. 10.1080/10717544.2018.1494223. 30394123 10.1080/10717544.2018.1494223 PMC6225487

[28] Straehla JP, et al. A predictive microfluidic model of human glioblastoma to assess trafficking of blood–brain barrier-penetrant nanoparticles. Proc Natl Acad Sci. 2022;119(23):e2118697119. 10.1073/pnas.2118697119. 35648828 10.1073/pnas.2118697119 PMC9191661

[29] Yang Z-Z. Tumor-targeting dual peptides-modified cationic liposomes for delivery of siRNA and docetaxel to gliomas. Biomaterials. 2014;35(19):5226–39. 10.1016/j.biomaterials.2014.03.017. 24695093 10.1016/j.biomaterials.2014.03.017

[30] Huang S, et al. Dual targeting effect of Angiopep-2-modified, DNA-loaded nanoparticles for glioma. Biomaterials. 2011;32(28):6832–8. 10.1016/j.biomaterials.2011.05.064. 21700333 10.1016/j.biomaterials.2011.05.064

[31] Zhu Y, et al. Highly efficacious and specific anti-glioma chemotherapy by tandem nanomicelles co-functionalized with brain tumor-targeting and cell-penetrating peptides. J Controlled Release. 2018;278:1–8. 10.1016/j.jconrel.2018.03.025. 10.1016/j.jconrel.2018.03.025 29596873

[32] Zhu Z, et al. Specific anti-glioma targeted-delivery strategy of engineered small extracellular vesicles dual-functionalised by Angiopep-2 and TAT peptides. J Extracell Vesicles. 2022;11(8):e12255. 10.1002/jev2.12255. 35932288 10.1002/jev2.12255 PMC9451528

[33] Gao H, Zhang S, Cao S, Yang Z, Pang Z, Jiang X. Angiopep-2 and Activatable Cell-Penetrating Peptide Dual-Functionalized Nanoparticles for Systemic Glioma-Targeting Delivery. Mol Pharm. 2014;11(8):2755–63. 10.1021/mp500113p. 24983928 10.1021/mp500113p

[34] Wei X, Zhan C, Chen X, Hou J, Xie C, Lu W. Retro-Inverso Isomer of Angiopep-2: A Stable d-Peptide Ligand Inspires Brain-Targeted Drug Delivery. Mol Pharm. 2014;11(10):3261–8. 10.1021/mp500086e. 24673510 10.1021/mp500086e

[35] Huile G, et al. A cascade targeting strategy for brain neuroglial cells employing nanoparticles modified with angiopep-2 peptide and EGFP-EGF1 protein. Biomaterials. 2011;32(33):8669–75. 10.1016/j.biomaterials.2011.07.069. 21843903 10.1016/j.biomaterials.2011.07.069

[36] Dal Magro R, et al. ApoE-modified solid lipid nanoparticles: A feasible strategy to cross the blood-brain barrier. J Controlled Release. 2017;249:103–10. 10.1016/j.jconrel.2017.01.039. 10.1016/j.jconrel.2017.01.039 28153761

[37] Formicola B, et al. The synergistic effect of chlorotoxin-mApoE in boosting drug-loaded liposomes across the BBB. J Nanobiotechnology. 2019;17(1):115. 10.1186/s12951-019-0546-3. 31711496 10.1186/s12951-019-0546-3 PMC6844026

[38] Formicola B et al. Differential Exchange of Multifunctional Liposomes Between Glioblastoma Cells and Healthy Astrocytes via Tunneling Nanotubes. Front Bioeng Biotechnol. 2019;7, 10.3389/fbioe.2019.00403. 10.3389/fbioe.2019.00403 PMC6920177 31921808

[39] Balducci C, et al. Multifunctional Liposomes Reduce Brain β-Amyloid Burden and Ameliorate Memory Impairment in Alzheimer’s Disease Mouse Models. J Neurosci. 2014;34(42):14022–31. 10.1523/JNEUROSCI.0284-14.2014. 25319699 10.1523/JNEUROSCI.0284-14.2014 PMC4198543

[40] Mancini S, et al. Multifunctional liposomes delay phenotype progression and prevent memory impairment in a presymptomatic stage mouse model of Alzheimer disease. J Controlled Release. 2017;258:121–9. 10.1016/j.jconrel.2017.05.013. 10.1016/j.jconrel.2017.05.013 28501671

[41] Bana L, et al. Liposomes bi-functionalized with phosphatidic acid and an ApoE-derived peptide affect Aβ aggregation features and cross the blood–brain-barrier: Implications for therapy of Alzheimer disease. Nanomed Nanotechnol Biol Med. 2014;10(7):1583–90. 10.1016/j.nano.2013.12.001. 10.1016/j.nano.2013.12.001 24333591

[42] Kumar P, et al. Transvascular delivery of small interfering RNA to the central nervous system. Nature. 2007;448(7149):39–43. 10.1038/nature05901. 17572664 10.1038/nature05901

[43] Hampel H, et al. The β-Secretase BACE1 in Alzheimer’s Disease. Biol Psychiatry. 2020;89(8):745. 10.1016/j.biopsych.2020.02.001. 32223911 10.1016/j.biopsych.2020.02.001 PMC7533042

[44] Poewe W, et al. Parkinson disease. Nat Rev Dis Primer. 2017;3(1):1–21. 10.1038/nrdp.2017.13. 10.1038/nrdp.2017.13 28332488

[45] Jiang P, et al. RVG29 Peptide-Modified Exosomes Loaded with Mir-133b Mediate the RhoA-ROCK Pathway to Improve Motor and Neurological Symptoms in Parkinson’s Disease. ACS Biomater Sci Eng. 2024;10(5):3069–85. 10.1021/acsbiomaterials.3c01622. 38578110 10.1021/acsbiomaterials.3c01622

[46] Lee JH, Engler JA, Collawn JF, Moore BA. Receptor mediated uptake of peptides that bind the human transferrin receptor. Eur J Biochem. 2001;268(7):2004–12. 10.1046/j.1432-1327.2001.02073.x. 11277922 10.1046/j.1432-1327.2001.02073.x

[47] Sun X, et al. T7 Peptide-modified macrophage membrane-coated nanoplatform for enhanced glioma treatment. Eur J Pharm Biopharm. 2024;204:114527. 10.1016/j.ejpb.2024.114527. 39383975 10.1016/j.ejpb.2024.114527

[48] Wei X, et al. A Lysosome-Targeted Magnetic Nanotorquer Mechanically Triggers Ferroptosis for Breast Cancer Treatment. Adv Sci. 2024;11(9):2302093. 10.1002/advs.202302093. 10.1002/advs.202302093 PMC10916606 38095513

[49] Wang Z, et al. Enhanced anti-ischemic stroke of ZL006 by T7-conjugated PEGylated liposomes drug delivery system. Sci Rep. 2015;5(1):12651. 10.1038/srep12651. 26219474 10.1038/srep12651 PMC4518266

[50] Zhang W, et al. Differential expression of receptors mediating receptor-mediated transcytosis (RMT) in brain microvessels, brain parenchyma and peripheral tissues of the mouse and the human. Fluids Barriers CNS. 2020;17(1):47. 10.1186/s12987-020-00209-0. 32698806 10.1186/s12987-020-00209-0 PMC7376922

[51] Demeule, Michel et al. Identification and design of peptides as a new drug delivery system for the brain. J Pharmacol Exp Ther. 2008;324(3):1064–72. 10.1124/jpet.107.131318. 10.1124/jpet.107.131318 18156463

[52] Jiang Y, Zhang J, Meng F, Zhong Z. Apolipoprotein E Peptide-Directed Chimeric Polymersomes Mediate an Ultrahigh-Efficiency Targeted Protein Therapy for Glioblastoma. ACS Nano. 2018;12(11):11070–9. 10.1021/acsnano.8b05265. 30395440 10.1021/acsnano.8b05265

[53] Wang X, Ciraolo G, Morris R, Gruenstein E. Identification of a neuronal endocytic pathway activated by an apolipoprotein E (apoE) receptor binding peptide. Brain Res. 1997;778(1):6–15. 10.1016/s0006-8993(97)00877-9. 9462872 10.1016/s0006-8993(97)00877-9

[54] Re F, et al. Functionalization of liposomes with ApoE-derived peptides at different density affects cellular uptake and drug transport across a blood-brain barrier model. Nanomed Nanotechnol Biol Med. 2011;7(5):551–9. 10.1016/j.nano.2011.05.004. 10.1016/j.nano.2011.05.004 21658472

[55] Dyer CA, Curtiss LK. A synthetic peptide mimic of plasma apolipoprotein E that binds the LDL receptor. J Biol Chem. 1991;266(34):22803–6. 10.1016/S0021-9258(18)54425-2. 1744074

[56] Kato N, et al. Development of an apolipoprotein E mimetic peptide–lipid conjugate for efficient brain delivery of liposomes. Drug Deliv. 2023;30(1):2173333. 10.1080/10717544.2023.2173333. 36718920 10.1080/10717544.2023.2173333 PMC9891163

[57] Schmidt N, Mishra A, Lai GH, Wong GCL. Arginine-rich cell-penetrating peptides. FEBS Lett. 2010;584(9):1806–13. 10.1016/j.febslet.2009.11.046. 19925791 10.1016/j.febslet.2009.11.046

[58] Sharma G, et al. Cell penetrating peptide tethered bi-ligand liposomes for delivery to brain in vivo: Biodistribution and transfection. J Controlled Release. 2013;167(1):1–10. 10.1016/j.jconrel.2013.01.016. 10.1016/j.jconrel.2013.01.016 23352910

[59] Cavaco M, et al. Molecular determinants for brain targeting by peptides: a meta-analysis approach with experimental validation. Fluids Barriers CNS. 2024;21(1):45. 10.1186/s12987-024-00545-5. 38802930 10.1186/s12987-024-00545-5 PMC11131246

[60] Chugh A, Eudes F. Translocation and nuclear accumulation of monomer and dimer of HIV-1 Tat basic domain in triticale mesophyll protoplasts. Biochim Biophys Acta BBA - Biomembr. 2007;1768(3):419–26. 10.1016/j.bbamem.2006.11.012. 10.1016/j.bbamem.2006.11.012 17214959

[61] Cheng Y, et al. Blood-Brain Barrier Permeable Gold Nanoparticles: An Efficient Delivery Platform for Enhanced Malignant Glioma Therapy and Imaging. Small. 2014;10(24):5137–50. 10.1002/smll.201400654. 25104165 10.1002/smll.201400654 PMC4268041

[62] Sharma G, Modgil A, Zhong T, Sun C, Singh J. Influence of Short-Chain Cell-Penetrating Peptides on Transport of Doxorubicin Encapsulating Receptor-Targeted Liposomes Across Brain Endothelial Barrier. Pharm Res. 2014;31(5):1194–209. 10.1007/s11095-013-1242-x. 24242938 10.1007/s11095-013-1242-x

[63] Shadmani N, et al. Enhancing Methotrexate Delivery in the Brain by Mesoporous Silica Nanoparticles Functionalized with Cell-Penetrating Peptide using in Vivo and ex Vivo Monitoring. Mol Pharm. 2023;20(3):1531–48. 10.1021/acs.molpharmaceut.2c00755. 36763486 10.1021/acs.molpharmaceut.2c00755

[64] Srimanee A, Arvanitidou M, Kim K, Hällbrink M, Langel Ü. Cell-penetrating peptides for siRNA delivery to glioblastomas. Peptides. 2018;104:62–9. 10.1016/j.peptides.2018.04.015. 29684592 10.1016/j.peptides.2018.04.015

[65] Shi K, et al. Liposomes Combined an Integrin αvβ3-Specific Vector with pH-Responsible Cell-Penetrating Property for Highly Effective Antiglioma Therapy through the Blood-Brain Barrier. ACS Appl Mater Interfaces. 2015;7(38):21442–54. 10.1021/acsami.5b06429. 26371468 10.1021/acsami.5b06429

[66] Liu Y, et al. Dual Receptor Recognizing Cell Penetrating Peptide for Selective Targeting, Efficient Intratumoral Diffusion and Synthesized Anti-Glioma Therapy. Theranostics. 2016;6(2):177–91. 10.7150/thno.13532. 26877777 10.7150/thno.13532 PMC4729767

[67] Teesalu T, Sugahara KN, Kotamraju VR, Ruoslahti E. C-end rule peptides mediate neuropilin-1-dependent cell, vascular, and tissue penetration. Proc Natl Acad Sci. 2009;106(38):16157–62. 10.1073/pnas.0908201106. 19805273 10.1073/pnas.0908201106 PMC2752543

[68] Liu Y, et al. Paclitaxel loaded liposomes decorated with a multifunctional tandem peptide for glioma targeting. Biomaterials. 2014;35(17):4835–47. 10.1016/j.biomaterials.2014.02.031. 24651033 10.1016/j.biomaterials.2014.02.031

[69] Wu C-H, Liu I-J, Lu R-M, Wu H-C. Advancement and applications of peptide phage display technology in biomedical science. J Biomed Sci. 2016;23(1):8. 10.1186/s12929-016-0223-x. 26786672 10.1186/s12929-016-0223-x PMC4717660

[70] van Rooy I, et al. Identification of Peptide Ligands for Targeting to the Blood-Brain Barrier. Pharm Res. 2010;27(4):673. 10.1007/s11095-010-0053-6. 20162339 10.1007/s11095-010-0053-6 PMC2837178

[71] Wu L-P, et al. Crossing the blood-brain-barrier with nanoligand drug carriers self-assembled from a phage display peptide. Nat Commun. 2019;10(1):4635. 10.1038/s41467-019-12554-2. 31604928 10.1038/s41467-019-12554-2 PMC6789111

[72] Mann AP, et al. A peptide for targeted, systemic delivery of imaging and therapeutic compounds into acute brain injuries. Nat Commun. 2016;7(1):11980. 10.1038/ncomms11980. 27351915 10.1038/ncomms11980 PMC4931241

[73] Yamaguchi S, Ito S, Kurogi-Hirayama M, Ohtsuki S. Identification of cyclic peptides for facilitation of transcellular transport of phages across intestinal epithelium *in vitro* and *in vivo*. J Controlled Release. 2017;262:232–8. 10.1016/j.jconrel.2017.07.037. 10.1016/j.jconrel.2017.07.037 28757359

[74] Yamaguchi S, Ito S, Masuda T, Couraud P-O, Ohtsuki S. Novel cyclic peptides facilitating transcellular blood-brain barrier transport of macromolecules *in vitro* and *in vivo*. J Controlled Release. 2020;321:744–55. 10.1016/j.jconrel.2020.03.001. 10.1016/j.jconrel.2020.03.001 32135226

[75] Guo Q, et al. A dual-ligand fusion peptide improves the brain-neuron targeting of nanocarriers in Alzheimer’s disease mice. J Controlled Release. 2020;320:347–62. 10.1016/j.jconrel.2020.01.039. 10.1016/j.jconrel.2020.01.039 31978446

[76] Zhang M, Lu W. Enhanced glioma-targeting and stability of L GICP peptide coupled with stabilized peptide D A7R. Acta Pharm Sin B. 2018;8(1):106–15. 10.1016/j.apsb.2017.11.004. 29872627 10.1016/j.apsb.2017.11.004 PMC5985625

[77] Saravanan R, et al. Structure, activity and interactions of the cysteine deleted analog of tachyplesin-1 with lipopolysaccharide micelle: Mechanistic insights into outer-membrane permeabilization and endotoxin neutralization. Biochim Biophys Acta BBA - Biomembr. 2012;1818(7):1613–24. 10.1016/j.bbamem.2012.03.015. 10.1016/j.bbamem.2012.03.015 22464970

[78] Crasto CJ, Feng J. LINKER: a program to generate linker sequences for fusion proteins. Protein Eng Des Sel. 2000;13(5):309–12. 10.1093/protein/13.5.309. 10.1093/protein/13.5.309 10835103

[79] Guo J, et al. Link-INVENT: generative linker design with reinforcement learning. Digit Discov. 2023;2(2):392–408. 10.1039/D2DD00115B.

[80] Ji X, Nielsen AL, Heinis C. Cyclic Peptides for Drug Development. Angew Chem. 2024;136(3):e202308251. 10.1002/ange.202308251. 10.1002/anie.202308251 37870189

[81] Malde AK, Hill TA, Iyer A, Fairlie DP. Crystal Structures of Protein-Bound Cyclic Peptides. Chem Rev. 2019;119(17):9861–914. 10.1021/acs.chemrev.8b00807. 31046237 10.1021/acs.chemrev.8b00807

[82] Corbett KM, Ford L, Warren DB, Pouton CW, Chalmers DK. Cyclosporin Structure and Permeability: From A to Z and Beyond. J Med Chem. 2021;64(18):13131–51. 10.1021/acs.jmedchem.1c00580. 34478303 10.1021/acs.jmedchem.1c00580

[83] Zhan C, Gu B, Xie C, Li J, Liu Y, Lu W. Cyclic RGD conjugated poly(ethylene glycol)-co-poly(lactic acid) micelle enhances paclitaxel anti-glioblastoma effect. J Controlled Release. 2010;143(1):136–42. 10.1016/j.jconrel.2009.12.020. 10.1016/j.jconrel.2009.12.020 20056123

[84] Belhadj Z, et al. Design of Y-shaped targeting material for liposome-based multifunctional glioblastoma-targeted drug delivery. J Controlled Release. 2017;255:132–41. 10.1016/j.jconrel.2017.04.006. 10.1016/j.jconrel.2017.04.006 28390902

[85] Miura Y, et al. Cyclic RGD-Linked Polymeric Micelles for Targeted Delivery of Platinum Anticancer Drugs to Glioblastoma through the Blood-Brain Tumor Barrier. ACS Nano. 2013;7(10):8583–92. 10.1021/nn402662d. 24028526 10.1021/nn402662d

[86] Chen C, et al. Peptide-22 and Cyclic RGD Functionalized Liposomes for Glioma Targeting Drug Delivery Overcoming BBB and BBTB. ACS Appl Mater Interfaces. 2017;9(7):5864–73. 10.1021/acsami.6b15831. 28128553 10.1021/acsami.6b15831

[87] Sakamoto K, et al. Cyclic Peptides KS-133 and KS-487 Multifunctionalized Nanoparticles Enable Efficient Brain Targeting for Treating Schizophrenia. JACS Au. 2024;4(8):2811–7. 10.1021/jacsau.4c00311. 39211592 10.1021/jacsau.4c00311 PMC11350716

[88] Sakamoto K. Generation of KS-487 as a novel LRP1-binding cyclic peptide with higher affinity, higher stability and BBB permeability. Biochem Biophys Rep. 2022;32:101367. 10.1016/j.bbrep.2022.101367. 36237444 10.1016/j.bbrep.2022.101367 PMC9552116

[89] Vacic V, et al. Duplications of the neuropeptide receptor gene VIPR2 confer significant risk for schizophrenia. Nature. 2011;471(7339):499–503. 10.1038/nature09884. 21346763 10.1038/nature09884 PMC3351382

[90] Bechtler C, Lamers C. Macrocyclization strategies for cyclic peptides and peptidomimetics. RSC Med Chem. 2021;12(8):1325–51. 10.1039/D1MD00083G. 34447937 10.1039/d1md00083g PMC8372203

[91] Taylor EM, Otero DA, Banks WA, O’Brien JS. Retro-inverso prosaptide peptides retain bioactivity, are stable in vivo, and are blood-brain barrier permeable. J Pharmacol Exp Ther. 2000;295(1):190–4. 10991978

[92] Brugidou J, Legrand C, Mery J, Rabie A. The *Retro-inverso* Form of a Homeobox-Derived Short Peptide Is Rapidly Internalized by Cultured Neurons: A New Basis for an Efficient Intracellular Delivery System. Biochem Biophys Res Commun. 1995;214(2):685–93. 10.1006/bbrc.1995.2340. 7677782 10.1006/bbrc.1995.2340

[93] Clark AJ, Davis ME. Increased brain uptake of targeted nanoparticles by adding an acid-cleavable linkage between transferrin and the nanoparticle core. Proc Natl Acad Sci. 2015;112(40):12486–91. 10.1073/pnas.1517048112. 26392563 10.1073/pnas.1517048112 PMC4603510

[94] Xiao W, et al. The protein corona hampers the transcytosis of transferrin-modified nanoparticles through blood–brain barrier and attenuates their targeting ability to brain tumor. Biomaterials. 2021;274:120888. 10.1016/j.biomaterials.2021.120888. 34029915 10.1016/j.biomaterials.2021.120888

[95] Zhang Z-A, et al. Novel brain-targeted nanomicelles for anti-glioma therapy mediated by the ApoE-enriched protein corona in vivo. J Nanobiotechnology. 2021;19(1):453. 10.1186/s12951-021-01097-8. 34963449 10.1186/s12951-021-01097-8 PMC8715648

[96] Pustulka SM, Ling K, Pish SL, Champion JA. Protein Nanoparticle Charge and Hydrophobicity Govern Protein Corona and Macrophage Uptake. ACS Appl Mater Interfaces. 2020;12(43):48284–95. 10.1021/acsami.0c12341. 33054178 10.1021/acsami.0c12341

[97] Su G, Jiang H, Xu B, Yu Y, Chen X. Effects of Protein Corona on Active and Passive Targeting of Cyclic RGD Peptide-Functionalized PEGylation Nanoparticles. Mol Pharm. 2018;15(11):5019–30. 10.1021/acs.molpharmaceut.8b00612. 30222356 10.1021/acs.molpharmaceut.8b00612

[98] Muttenthaler M, King GF, Adams DJ, Alewood PF. Trends in peptide drug discovery. Nat Rev Drug Discov. 2021;20(4):309–25. 10.1038/s41573-020-00135-8. 33536635 10.1038/s41573-020-00135-8

[99] Vinogradov AA, Yin Y, Suga H. Macrocyclic Peptides as Drug Candidates: Recent Progress and Remaining Challenges. J Am Chem Soc. 2019;141(10):4167–81. 10.1021/jacs.8b13178. 30768253 10.1021/jacs.8b13178

[100] Notin P, Rollins N, Gal Y, Sander C, Marks D. Machine learning for functional protein design. Nat Biotechnol. 2024;42(2):216–28. 10.1038/s41587-024-02127-0. 38361074 10.1038/s41587-024-02127-0 PMC13159571

[101] Weininger D. SMILES, a chemical language and information system. 1. Introduction to methodology and encoding rules. J Chem Inf Model. 1988; 28(1):31–6. 10.1021/ci00057a005.

[102] Vaswani A, et al. Attention is all you need. Adv Neural Inf Process Syst. 2017;30. 10.48550/arXiv.1706.03762.

[103] Lin Z, et al. Evolutionary-scale prediction of atomic-level protein structure with a language model. 2023;379:1123–30. 10.1126/science.ade2574. 10.1126/science.ade2574 36927031

[104] UniProt: the universal protein knowledgebase in 2023. Nucleic Acids Res. 2023;51(D1):D523–D531. 10.1093/nar/gkac1052. 10.1093/nar/gkac1052 PMC9825514 36408920

[105] Chen T, Vure P, Pulugurta R, Chatterjee P. AMP-Diffusion: integrating latent diffusion with protein language models for antimicrobial peptide generation. bioRxiv. 2024;583201. 10.1101/2024.03.03.583201.

[106] Bhat S et al. De Novo Generation and Prioritization of Target-Binding Peptide Motifs from Sequence Alone. bioRxiv. 2023. 10.1101/2023.06.26.546591.

[107] Bhat S et al. De Novo Design of Peptide Binders to Conformationally Diverse Targets with Contrastive Language Modeling. bioRxiv 2024. 10.1101/2023.06.26.546591. 10.1126/sciadv.adr8638 PMC11753435 39841846

[108] Brixi G, et al. SaLT&PepPr is an interface-predicting language model for designing peptide-guided protein degraders. Commun Biol. 2023;6(1):1–10. 10.1038/s42003-023-05464-z. 37875551 10.1038/s42003-023-05464-z PMC10598214

[109] Watson JL, et al. De novo design of protein structure and function with RFdiffusion. Nature. 2023;620(7976):1089–100. 10.1038/s41586-023-06415-8. 37433327 10.1038/s41586-023-06415-8 PMC10468394

[110] Dauparas J, et al. Robust deep learning–based protein sequence design using ProteinMPNN. Science. 2022;378:49–56. 10.1126/science.add2187. 36108050 10.1126/science.add2187 PMC9997061

[111] Hsu C et al. Learning inverse folding from millions of predicted structures. 2022. 10.1101/2022.04.10.487779.

[112] Wang L, et al. Therapeutic peptides: current applications and future directions. Signal Transduct Target Ther. 2022;7(1):1–27. 10.1038/s41392-022-00904-4. 35165272 10.1038/s41392-022-00904-4 PMC8844085

[113] Chen T, Zhang Y, Chatterjee P. moPPIt: De Novo Generation of Motif-Specific Binders with Protein Language Models. bioRxiv. 2024. 10.1101/2024.07.31.606098.

[114] Rettie SA et al.. Accurate de novo design of high-affinity protein binding macrocycles using deep learning. bioRxiv. 2024. 10.1101/2024.11.18.622547. 10.1038/s41589-025-01929-w PMC12643943 40542165

[115] Choudhuri VSRS, Ghosh B. Hybrid Diffusion Model for Stable, Affinity-Driven, Receptor-Aware Peptide Generation. J Chem Inf Model. 2024;64(17):6912–25. 10.1021/acs.jcim.4c01020. 39193724 10.1021/acs.jcim.4c01020

[116] Baek M, Anishchenko I, Humphreys IR, Cong Q, Baker D, DiMaio F. Efficient and accurate prediction of protein structure using RoseTTAFold2. 2023, bioRxiv. 10.1101/2023.05.24.542179.

[117] Mirdita M, Schütze K, Moriwaki Y, Heo L, Ovchinnikov S, Steinegger M. ColabFold: making protein folding accessible to all. Nat Methods. 2022;19(6):679–82. 10.1038/s41592-022-01488-1. 35637307 10.1038/s41592-022-01488-1 PMC9184281

[118] Bayrak A, Hanson J, Laufer S, Pillaiyar T. Super-Conserved Receptors Expressed in the Brain: Biology and Medicinal Chemistry Efforts. Future Med Chem. 2022;14(12):899–913. 10.4155/fmc-2022-0006. 35535715 10.4155/fmc-2022-0006

[119] Huang ETC, et al. Predicting blood–brain barrier permeability of molecules with a large language model and machine learning. Sci Rep. 2024;14(1):15844. 10.1038/s41598-024-66897-y. 38982309 10.1038/s41598-024-66897-y PMC11233737

[120] Kumar R, Sharma A, Alexiou A, Bilgrami AL, Kamal MA, Ashraf GM. DeePred-BBB: A Blood Brain Barrier Permeability Prediction Model With Improved Accuracy. Front Neurosci. 2022, 10.3389/fnins.2022.858126. 10.3389/fnins.2022.858126 PMC9112838 35592264

[121] Shaker B, et al. LightBBB: computational prediction model of blood–brain-barrier penetration based on LightGBM. Bioinformatics. 2021;37(8):1135–9. 10.1093/bioinformatics/btaa918. 33112379 10.1093/bioinformatics/btaa918

[122] Liu L, et al. Prediction of the Blood-Brain Barrier (BBB) Permeability of Chemicals Based on Machine-Learning and Ensemble Methods. Chem Res Toxicol. 2021;34(6):1456–67. 10.1021/acs.chemrestox.0c00343. 34047182 10.1021/acs.chemrestox.0c00343

[123] Pereira T, Abbasi M, Oliveira JL, Ribeiro B, Arrais J. Optimizing blood–brain barrier permeation through deep reinforcement learning for de novo drug design. Bioinformatics. 2021;37(Supplement_1):i84–i92. 10.1093/bioinformatics/btab301. 10.1093/bioinformatics/btab301 PMC8336597 34252946

[124] Tang S, Zhang Y, Chatterjee P. PepTune: De novo generation of therapeutic peptides with multi-objective-guided discrete diffusion. Arxiv. 2025;2412.17780 . 10.48550/arXiv.2412.17780.

[125] Chen T et al. PepMLM: Target Sequence-Conditioned Generation of Therapeutic Peptide Binders via Span Masked Language Modeling. 2024, arXiv: arXiv:2310.03842. 10.48550/arXiv.2310.03842.

[126] Bhat S et al. De novo design of peptide binders to conformationally diverse targets with contrastive language modeling. Sci Adv. 2025;11:eadr8638. 10.1126/sciadv.adr8638 10.1126/sciadv.adr8638 PMC11753435 39841846

[127] Radford A et al. Learning Transferable Visual Models From Natural Language Supervision. 2021, arXiv: arXiv:2103.00020. 10.48550/arXiv.2103.00020.

[128] Abramson J, et al. Accurate structure prediction of biomolecular interactions with AlphaFold 3. Nature. 2024;630(8016):493–500. 10.1038/s41586-024-07487-w. 38718835 10.1038/s41586-024-07487-w PMC11168924

[129] Chen Q, et al. SREB2/GPR85, a schizophrenia risk factor, negatively regulates hippocampal adult neurogenesis and neurogenesis-dependent learning and memory. Eur J Neurosci. 2012;36(5):2597–608. 10.1111/j.1460-9568.2012.08180.x. 22697179 10.1111/j.1460-9568.2012.08180.x PMC3466408

[130] Radulescu E, et al. Effect of Schizophrenia Risk-Associated Alleles in SREB2 (GPR85) on Functional MRI Phenotypes in Healthy Volunteers. Neuropsychopharmacology. 2013;38(2):341–9. 10.1038/npp.2012.184. 22968816 10.1038/npp.2012.184 PMC3527120

[131] Fujita-Jimbo E, et al. The association of GPR85 with PSD-95-neuroligin complex and autism spectrum disorder: a molecular analysis. Mol Autism. 2015;6(1):17. 10.1186/s13229-015-0012-5. 25780553 10.1186/s13229-015-0012-5 PMC4360946

[132] Larco DO, Semsarzadeh NN, Cho-Clark M, Mani SK, John Wu T. The Novel Actions of the Metabolite GnRH-(1–5) are Mediated by a G Protein-Coupled Receptor. Front Endocrinol. 2013;4:83. 10.3389/fendo.2013.00083. 10.3389/fendo.2013.00083 PMC3703583 23847594

[133] Mcilwraith EK, Belsham DD. Phoenixin: uncovering its receptor, signaling and functions. Acta Pharmacol Sin. 2018;39(5):774–8. 10.1038/aps.2018.13. 29671415 10.1038/aps.2018.13 PMC5943909

[134] Martin AL, Steurer MA, Aronstam RS. Constitutive Activity among Orphan Class-A G Protein Coupled Receptors. PLoS ONE. 2015;10(9):e0138463. 10.1371/journal.pone.0138463. 26384023 10.1371/journal.pone.0138463 PMC4575141

[135] Swetha K, et al. Recent Advances in the Lipid Nanoparticle-Mediated Delivery of mRNA Vaccines. Vaccines. 2023;11(3):658. 10.3390/vaccines11030658. 36992242 10.3390/vaccines11030658 PMC10059764

[136] Metselaar JM, Lammers T. Challenges in nanomedicine clinical translation. Drug Deliv Transl Res. 2020;10(3):721–5. 10.1007/s13346-020-00740-5. 32166632 10.1007/s13346-020-00740-5 PMC7228980

[137] Collins JM, Porter KA, Singh SK, Vanier GS. High-Efficiency Solid Phase Peptide Synthesis (HE-SPPS). Org Lett. 2014;16(3):940–3. 10.1021/ol4036825. 24456219 10.1021/ol4036825

